# Supplementary material for: A General Strategy for Discovery of Inhibitors and Activators of RING and U-box E3 Ligases with Ubiquitin Variants
Source: Mol Cell. 2017 Oct 19;68(2):456–470.e10. doi: 10.1016/j.molcel.2017.09.027 (PMC5655547; doi:10.1016/j.molcel.2017.09.027)
Supplement: Document S1. Figures S1–S6 [file mmc1.pdf]

Molecular Cell, Volume *68*

Supplemental Information

A General Strategy for Discovery of Inhibitors and Activators of RING and U-box E3 Ligases with Ubiquitin Variants

Mads Gabrielsen, Lori Buetow, Mark A. Nakasone, Syed Feroj Ahmed, Gary J. Sibbet, Brian O. Smith, Wei Zhang, Sachdev S. Sidhu, and Danny T. Huang

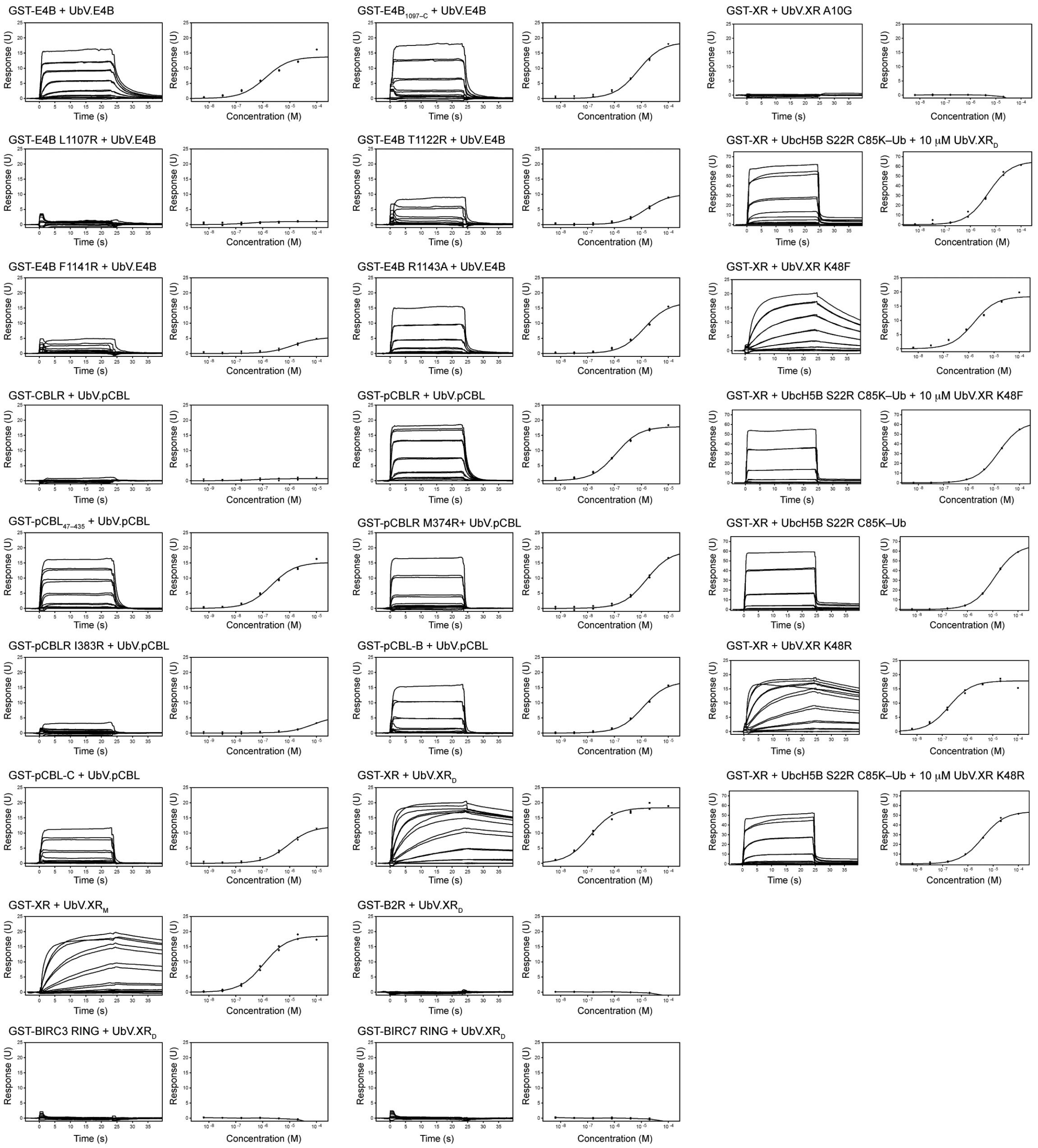

Figure S1 SPR analyses of GST-E3s and analyte binding affinities, related to Table 1. Representative sensorgrams (left) and binding curves (right) for GST-RING or U-box domains and their respective analytes as indicated above each set of sensorgram and binding curve. For GST-XR + UbcH5B S22R C85K–Ub + UbV.XR_D, UbV.XR K48R and UbV.XR K48F analyses, the concentration of UbcH5B S22R C85K–Ub was varied while the concentration of each UbV.XR variant was maintained at 10 μM. n=2 for each binding curve.

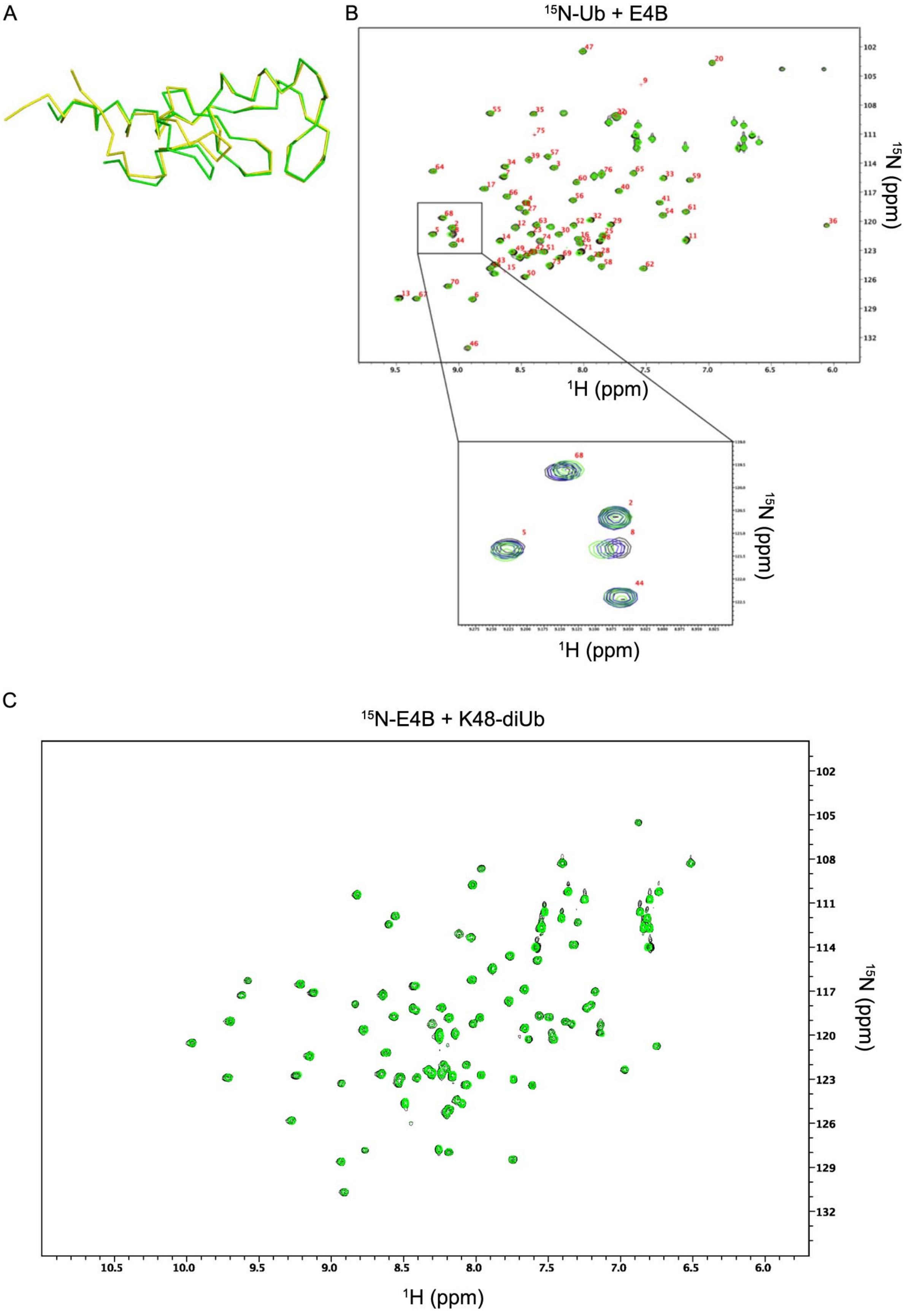
A
B
15N-Ub + E4B
1H (ppm)
15N (ppm)
1H (ppm)
15N (ppm)
C
15N-E4B + K48-diUb
1H (ppm)
15N (ppm)

Figure S2 Analyses of E4B structure and binding to Ub to Figure 2. (**A**) Overlay of $E4B_{1097-C}$ (green) with UBE4B (yellow) from PDBID 3L1Z (r.m.s.d. 0.631 Å across 431 atoms). (**B**) Chemical shift perturbation data for Ub-E4B interactions. ^{1}H-^{15}N HSQC spectra for ^{15}N-Ub alone (black) and with E4B (green, [^{15}N-Ub]:[E4B]=4.98). Inset shows close-up of selected peaks of ^{15}N-Ub alone (black) and with E4B (blue, [^{15}N-Ub]:[E4B]=1.29; green, [^{15}N-Ub]:[E4B]=4.98). (**C**) Chemical shift perturbation data for K48-diUb interactions with E4B. ^{1}H-^{15}N HSQC spectra for ^{15}N-E4B alone (black) and with K48-diUb (green, [K48-diUb]:[^{15}N-E4B]=1.87).

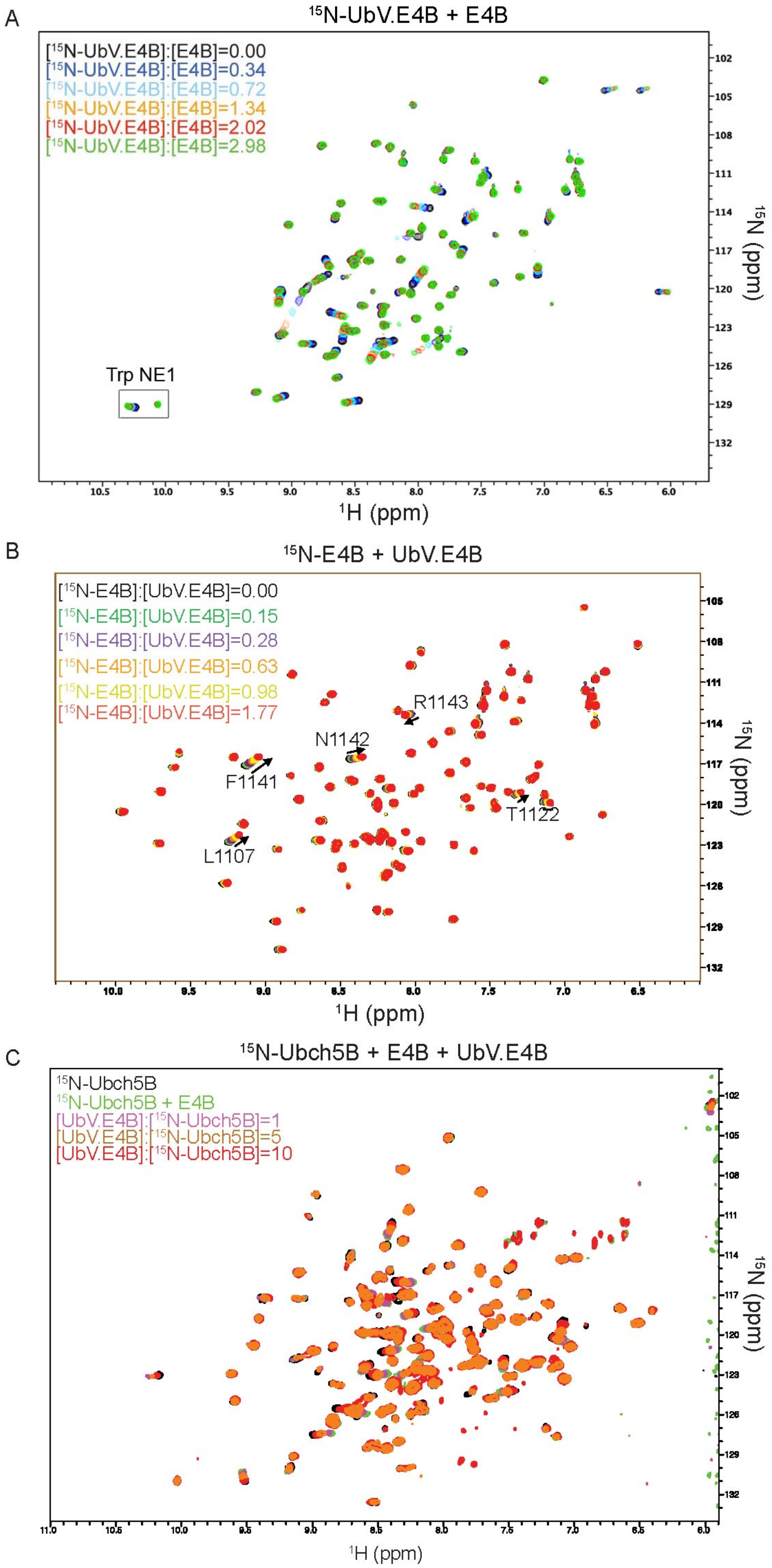
A
15N-UbV.E4B + E4B
[15N-UbV.E4B]:[E4B]=0.00
[15N-UbV.E4B]:[E4B]=0.34
[15N-UbV.E4B]:[E4B]=0.72
[15N-UbV.E4B]:[E4B]=1.34
[15N-UbV.E4B]:[E4B]=2.02
[15N-UbV.E4B]:[E4B]=2.98
Trp NE1
1H (ppm)
15N (ppm)
B
15N-E4B + UbV.E4B
[15N-E4B]:[UbV.E4B]=0.00
[15N-E4B]:[UbV.E4B]=0.15
[15N-E4B]:[UbV.E4B]=0.28
[15N-E4B]:[UbV.E4B]=0.63
[15N-E4B]:[UbV.E4B]=0.98
[15N-E4B]:[UbV.E4B]=1.77
R1143
N1142
F1141
T1122
L1107
1H (ppm)
15N (ppm)
C
15N-Ubch5B + E4B + UbV.E4B
15N-Ubch5B
15N-Ubch5B + E4B
[UbV.E4B]:[15N-Ubch5B]=1
[UbV.E4B]:[15N-Ubch5B]=5
[UbV.E4B]:[15N-Ubch5B]=10
1H (ppm)
15N (ppm)

Figure S3 Analyses of E4B binding to UbV.E4B, related to Figure 2. (**A**) Chemical shift perturbation data for UbV.E4B in UbV.E4B-E4B interactions. ^{1}H-^{15}N HSQC spectra for ^{15}N-UbV.E4B alone (black) and with selected molar ratios of E4B indicated by different colors. A 281 μM sample of ^{15}N-UbV.E4B was titrated with a 3.65 mM solution of E4B until a molar ratio of 3:1 was reached. At the endpoint, the concentrations of ^{15}N-UbV.E4B and E4B were 228 μM and 682 μM, respectively. (**B**) Chemical shift perturbation data for E4B in UbV.E4B-E4B interactions. ^{1}H-^{15}N HSQC spectra for ^{15}N-E4B alone (black) and with selected molar ratios of UbV.E4B indicated by different colors. A 150 μM sample of ^{15}N-E4B was titrated with a 355 μM solution of UbV.E4B until a molar ratio of 1.77:1 was achieved. At the endpoint, the concentrations of ^{15}N-E4B and UbV.E4B were 86 μM and 152 μM, respectively. (**C**) Chemical shift perturbation data for UbcH5B in competition assays with UbV.E4B for binding to E4B. ^{1}H-^{15}N HSQC spectra for ^{15}N-UbcH5B alone (black, 150 μM), following addition of a one molar equivalent of a 3.5 mM solution of E4B (green) and subsequently titrated with selected molar ratios of a 0.355 mM solution of UbV.E4B indicated by different colors until [UbV.E4B]:[^{15}N-UbcH5B] was 10:1. At the endpoint, the concentrations of ^{15}N-UbcH5B, E4B and UbV.E4B were 30, 30 and 300 μM, respectively.

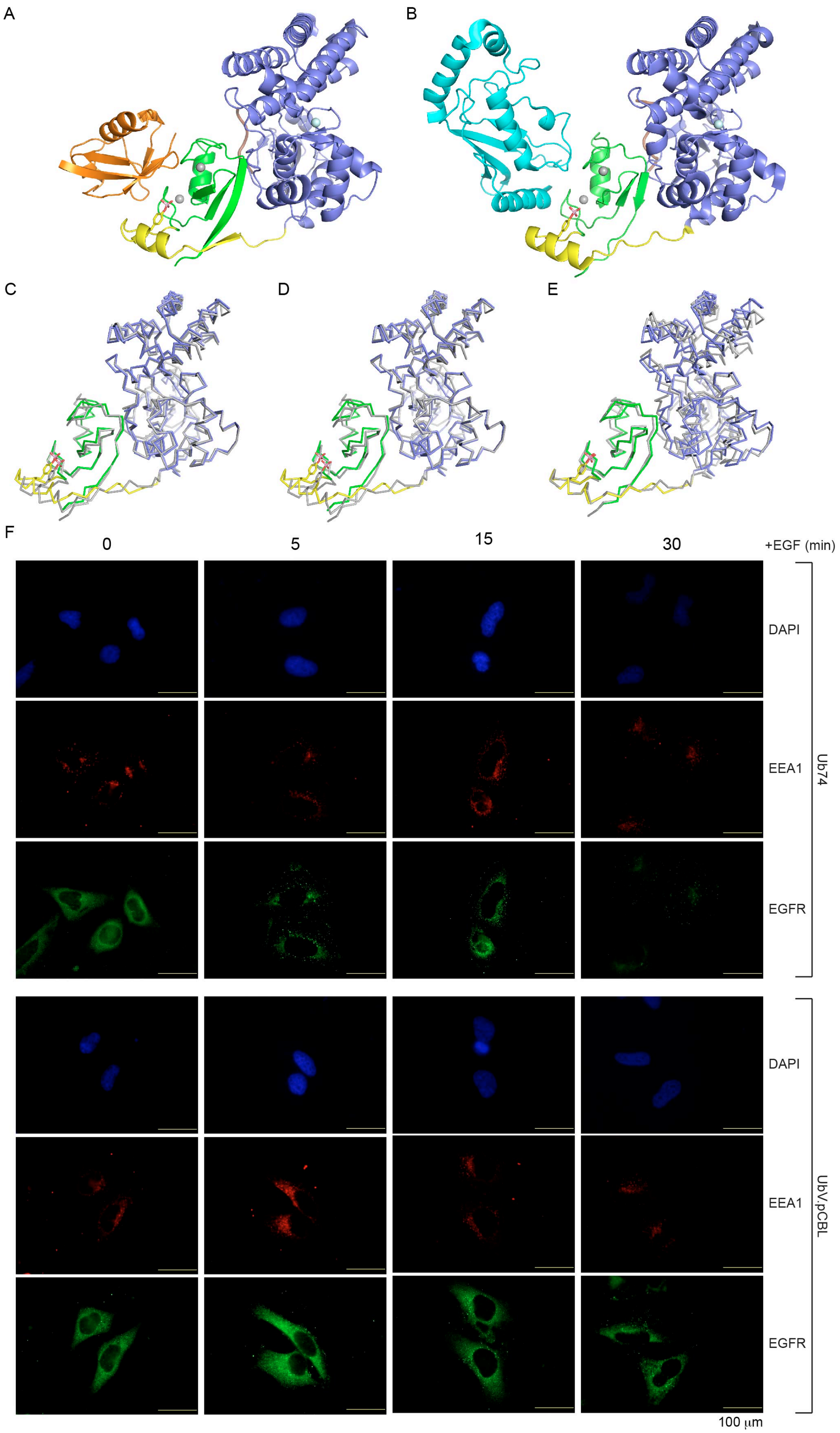
A
B
C
D
E
F
0
5
15
30
+EGF (min)
DAPI
EEA1
EGFR
Ub74
DAPI
EEA1
EGFR
UbV.pCBL
100 μm

Figure S4 UbV.pCBL effects on pCBL, related to Figure 3. (**A**) Cartoon representation of ZAP70 peptide-pCBL$_{47–435}$-UbV.pCBL colored as in **Figure 3** with pTyr371 shown in sticks. (**B)** Cartoon representation of ZAP70 peptide-pCBL$_{47–435}$-UbcH5B (PDBID 4A4B) with ZAP70 peptide and pCBL$_{47–435}$ colored as in **Figure 3** and UbcH5B colored cyan. pTyr371 is shown in sticks. **(C)** Ribbon overlay of pCBL$_{47–435}$ from the complexes shown in **A** and **B** (r.m.s.d. 1.37 Å across 379 Cα atoms). **(D)** Ribbon overlay based on the TKBD of pCBL$_{47–435}$ from the complexes shown in **A** and **B** (r.m.s.d. 1.21 Å across 303 Cα atoms). (**E**) Ribbon overlay based on the linker region and RING domain of pCBL$_{47–435}$ from the complexes shown in **A** and **B** (r.m.s.d. 0.64 Å across 74 Cα atoms). pCBL$_{47–435}$ is in the same orientation in all panels. For **C–E**, pCBL$_{47–435}$ from the UbcH5B-bound complex (**B**) is colored grey. (**F**) Images from HeLa cells overexpressing UbV.pCBL or Ub74 and treated with EGF as indicated. The cells were incubated with anti-EGFR and anti-EEA1 primary antibodies, followed by secondary antibodies conjugated to AF488 (EGFR, green) or AF594 (EEA1, red). DAPI was used to stain the nuclei. Scale bars in each panel represent 100 μm.

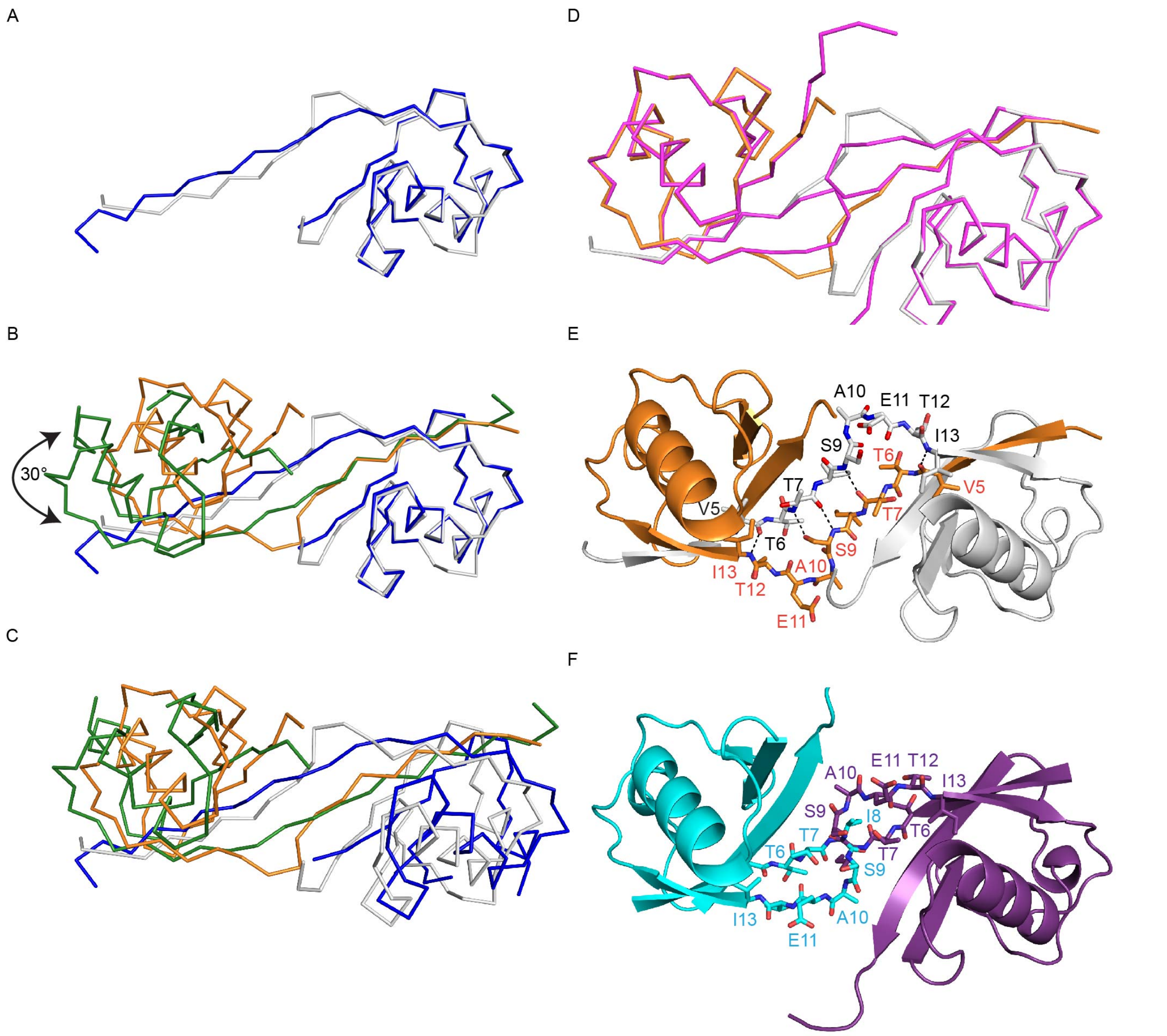

Figure S5 Comparison of UbV.XR dimers, related to Figure 5. (**A**) Ribbon overlay of a single subunit of UbV.XR$_D$ from the crystal structure of UbV.XR$_D$ alone (blue) onto UbV.XR$_D$ (white) from UbV.XR$_D$–XR complex (r.m.s.d. of 0.46 Å for 66 Cα atoms). (**B**) Ribbon overlay as in **A** but showing both subunits of UbV.XR$_D$ from the crystal structures of UbV.XR$_D$ alone (green and blue) and bound to XR (orange and white). The relative orientation of the two UbV.XR subunits differs by a 30° rotation. (**C**) Ribbon overlay of both subunits of UbV.XR$_D$ from the crystal structure of UbV.XR$_D$ alone (blue and white) onto UbV.XR$_D$ from the UbV.XR$_D$–XR complex (r.m.s.d. of 3.3 Å for 133 Cα atoms). (**D**) Ribbon overlay of Ub (magenta) onto both subunits of UbV.XR$_D$ from the UbV.XR$_D$–XR complex (r.m.s.d. of 0.4 Å for 72 Cα atoms, if β1' is treated as β1). (**E**) Close-up of UbV.XR$_D$ interface from the UbV.XR$_D$–XR complex. (**F**) Close-up of model of UbV.XR$_M$ dimer interface based on **D** in which wild-type Ub residues have been replaced with the corresponding residues in UbV.XR$_M$. One subunit is colored cyan and the other purple for clarity. Coloring is as described in **Figure 5**.

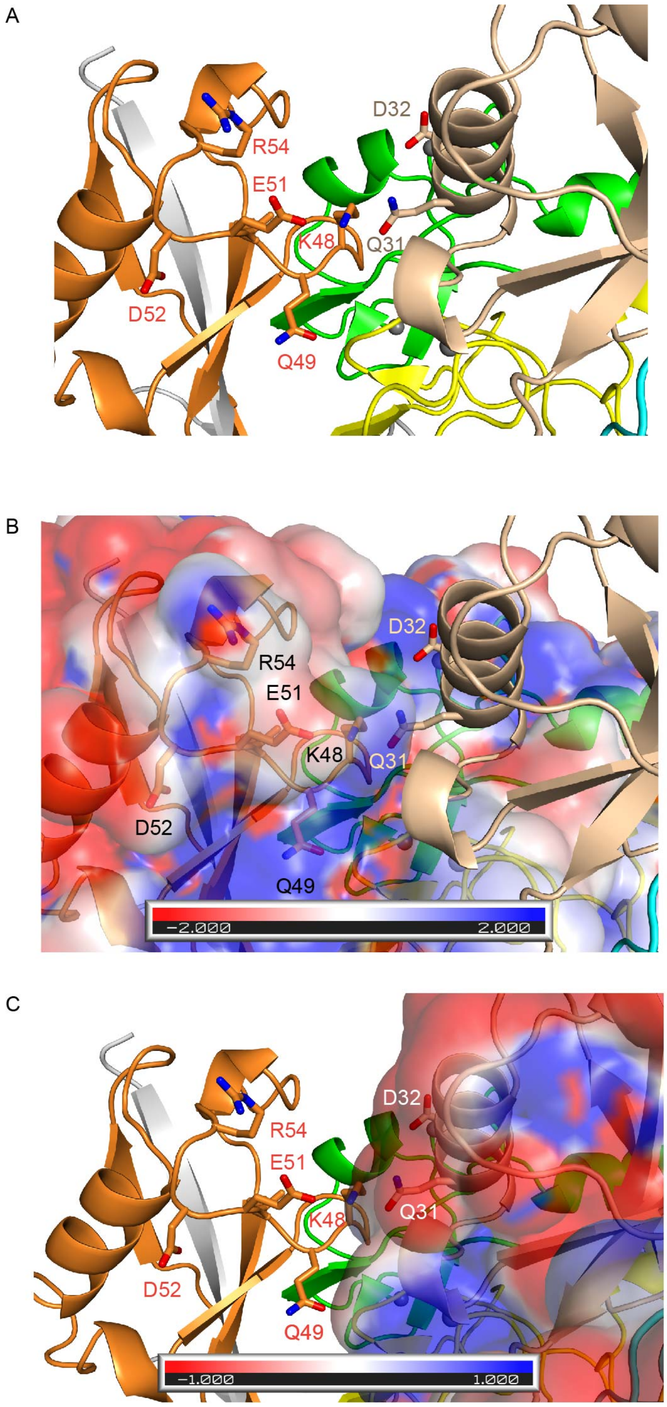

Figure S6 Mechanism of stabilization of E2~Ub by UbV.XR$_D$, related to Figure 5. (**A**) Close-up of donor Ub and UbV.XR$_D$ regions involved in electrostatic interactions from model of UbcH5B–Ub bound to the XR-UbV$_D$.XR complex. (**B**) As in **A** but including an electrostatic potential surface of the XR-UbV$_D$.XR complex. (**C**) As in **A** but including an electrostatic potential surface of UbcH5B–Ub from PDBID 4AUQ. Coloring is as described in **Figure 5**.
