# Supplementary material for: A General Strategy for Discovery of Inhibitors and Activators of RING and U-box E3 Ligases with Ubiquitin Variants
Source: Mol Cell. 2017 Oct 19;68(2):456–470.e10. doi: 10.1016/j.molcel.2017.09.027 (PMC5655547; doi:10.1016/j.molcel.2017.09.027)
Supplement: Document S2. Article plus Supplemental Information [file mmc2.pdf]

Resource

Molecular Cell

A General Strategy for Discovery of Inhibitors and Activators of RING and U-box E3 Ligases with Ubiquitin Variants

Graphical Abstract

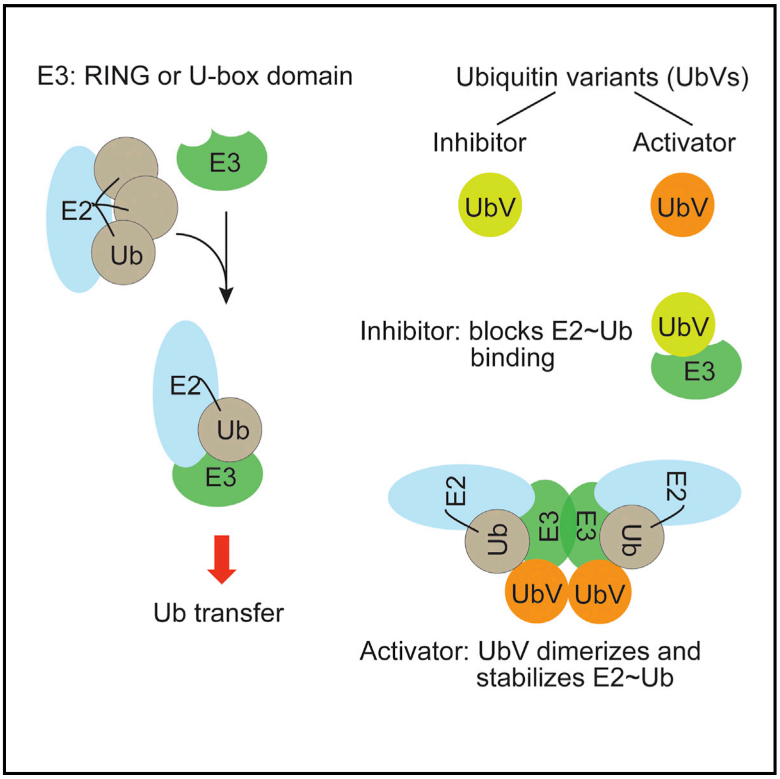

Highlights

- Identification of selective UbVs that inhibit or activate RING/U-box E3 ligases
- Structural and biochemical analyses reveal mechanisms of modulation by UbVs
- UbVs inhibit CBL and UBE4B by blocking the E2∼Ub binding site
- A UbV dimer stimulates XIAP activity by stabilizing E2∼Ub

Authors

Mads Gabrielsen, Lori Buetow, Mark A. Nakasone, ..., Wei Zhang, Sachdev S. Sidhu, Danny T. Huang

Correspondence

wx.zhang@mail.utoronto.ca (W.Z.),
sachdev.sidhu@utoronto.ca (S.S.S.),
d.huang@beatson.gla.ac.uk (D.T.H.)

In Brief

Gabrielsen et al. report the identification of selective ubiquitin variants that inhibit or activate the catalytic domains of RING/U-box E3 ligases. Inhibition is achieved by blocking the E2∼ubiquitin binding surface of RING/U-box domains, whereas activation is accomplished by stabilizing the primed E2∼ubiquitin conformation.

Gabrielsen et al., 2017, Molecular Cell *68*, 456–470
October 19, 2017 © 2017 The Author(s). Published by Elsevier Inc.
https://doi.org/10.1016/j.molcel.2017.09.027

CellPress

CellPress

Molecular Cell
Resource

A General Strategy for Discovery of Inhibitors and Activators of RING and U-box E3 Ligases with Ubiquitin Variants

Mads Gabrielsen,[1,5] Lori Buetow,[1,5] Mark A. Nakasone,[1] Syed Feroj Ahmed,[1] Gary J. Sibbet,[1] Brian O. Smith,[2] Wei Zhang,[3,*] Sachdev S. Sidhu,[3,*] and Danny T. Huang[1,4,6,*]
[1]Cancer Research UK Beatson Institute, Garscube Estate, Switchback Road, Glasgow G61 1BD, UK
[2]Institute of Molecular Cell and Systems Biology, University of Glasgow, Glasgow G12 8QQ, UK
[3]Donnelly Centre for Cellular and Biomolecular Research, Banting and Best Department of Medical Research, University of Toronto, 160 College Street, Toronto, ON M5S3E1, Canada
[4]Institute of Cancer Sciences, University of Glasgow, Glasgow G61 1BD, UK
[5]These authors contributed equally
[6]Lead Contact
*Correspondence: wx.zhang@mail.utoronto.ca (W.Z.), sachdev.sidhu@utoronto.ca (S.S.S.), d.huang@beatson.gla.ac.uk (D.T.H.)
https://doi.org/10.1016/j.molcel.2017.09.027

SUMMARY

RING and U-box E3 ubiquitin ligases regulate diverse eukaryotic processes and have been implicated in numerous diseases, but targeting these enzymes remains a major challenge. We report the development of three ubiquitin variants (UbVs), each binding selectively to the RING or U-box domain of a distinct E3 ligase: monomeric UBE4B, phosphorylated active CBL, or dimeric XIAP. Structural and biochemical analyses revealed that UbVs specifically inhibited the activity of UBE4B or phosphorylated CBL by blocking the E2∼Ub binding site. Surprisingly, the UbV selective for dimeric XIAP formed a dimer to stimulate E3 activity by stabilizing the closed E2∼Ub conformation. We further verified the inhibitory and stimulatory functions of UbVs in cells. Our work provides a general strategy to inhibit or activate RING/U-box E3 ligases and provides a resource for the research community to modulate these enzymes.

INTRODUCTION

Ubiquitination is a post-translational modification that is required for virtually all cellular processes (Hershko and Ciechanover, 1998). Ubiquitination involves the covalent attachment of the small protein ubiquitin (Ub) to N termini or lysines of proteins by the E1-E2-E3 enzyme cascade, and different Ub chains confer distinct functions on protein substrates (Komander and Rape, 2012; Pickart and Eddins, 2004). Ub ligases (E3s) mediate the final step of the process and control specificity, efficiency, and patterns of ubiquitination. Consequently, dysregulation of E3s occurs in many diseases, including diabetes, neurodegeneration, atherosclerosis, and cancer (Goru et al., 2016; Popovic et al., 2014), and E3s are attractive therapeutic targets (Nalepa et al., 2006; Petroski, 2008).

E3s are classified into three families: RING (really interesting new gene) and U-box E3s, HECT (homologous to E6AP C terminus) E3s, and RBR (RING between RING) E3s (Buetow and Huang, 2016). With ∼600 members, the RING/U-box E3s form the largest family (Deshaies and Joazeiro, 2009). This family uses a RING or U-box domain to recruit E2 enzymes thioesterified with Ub (E2∼Ub) and facilitates transfer of Ub directly to substrate. RING/U-box E3s can be further categorized as multisubunit complexes, which contain scaffold, adaptor, receptor, and RING subunits on distinct polypeptide chains (Deshaies and Joazeiro, 2009), or simple, which contain E2∼Ub and substrate-binding domains within the same polypeptide chain. Some simple RING/U-box E3s are active as monomers, like CBL and UBE4B, whereas others, like XIAP (X-linked inhibitor of apoptosis), only function when dimerized via their E2∼Ub binding domains (Buetow and Huang, 2016).

Despite the plethora of RING/U-box family members, small molecule modulators are limited to a few E3-substrate interactions (Bulatov and Ciulli, 2015; Landré et al., 2014). Substrate-binding domains vary among E3s; hence, new screening strategies are required to establish a general targeting platform. Although a RING or U-box domain is a common feature, these domains lack binding pockets easily targeted by inhibitors. Thus, we sought to develop a general platform to systematically modulate activities of RING and U-box E3s.

RING/U-box domains promote Ub transfer by shifting the equilibrium of E2∼Ub into a closed conformation (Dou et al., 2012b; Plechanovová et al., 2012; Pruneda et al., 2012). These domains comprise ∼75–100 aa that form two loops stabilized by two Zn^{2+} ions or hydrogen bonds in RING or U-box domains, respectively. These loops and intervening region form the E2∼Ub-binding surface in RING-E2∼Ub complexes, and the C-terminal tail and Ile36 patch of Ub form interactions with the RING while the Ile44 hydrophobic patch abuts the E2 (Buetow and Huang, 2016). Typically, a small surface area of only ∼450 Å^2 from the RING domain contacts Ub.

Previously, we used phage display to select for ubiquitin variants (UbVs) with enhanced affinities for proteins that naturally interact weakly with Ub (Ernst et al., 2013), including

 © 2017 The Author(s). Published by Elsevier Inc.
This is an open access article under the CC BY license (http://creativecommons.org/licenses/by/4.0/).

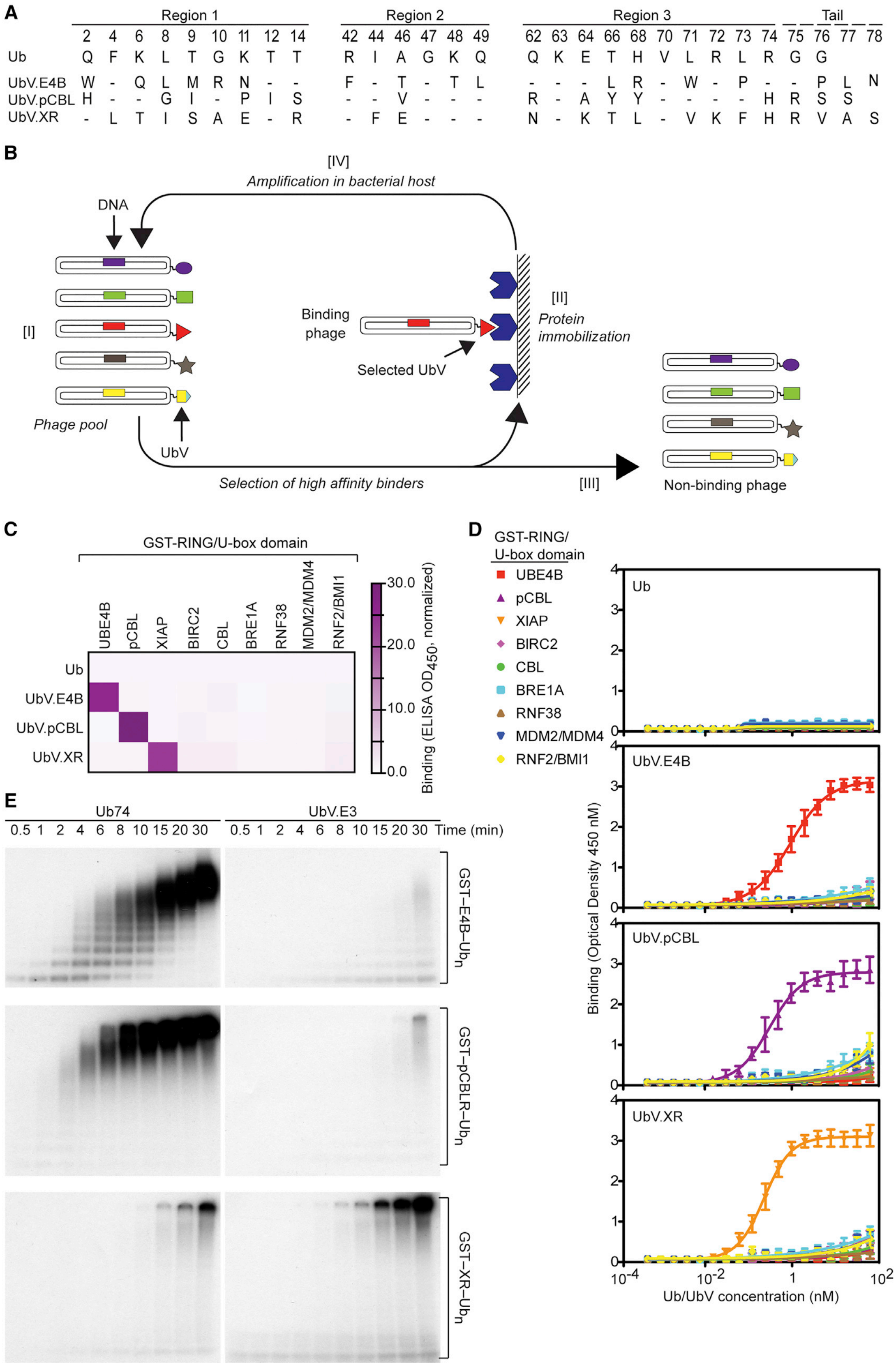

(legend on next page)

deubiquitinases, HECT E3s, multi-subunit E3s, and small Ub-interacting motifs (Brown et al., 2016; Ernst et al., 2013; Gorelik et al., 2016; Manczyk et al., 2017; Zhang et al., 2016, 2017a, 2017b). Here, we tested whether UbV modulators can be selected for simple RING E3s. We targeted three RING/U-box E3s from three important classes: (1) UBE4B, a monomeric U-box E3 (Wu et al., 2011); (2) CBL, a monomeric RING E3 activated by phosphorylation (Dou et al., 2012a); and (3) XIAP, a RING E3 that is activated upon dimerization (Nakatani et al., 2013). We generated selective UbVs for each RING/U-box domain and used biochemical assays and structural studies to identify two types of UbVs: competitive inhibitors of E2~Ub binding sites on UBE4B or phosphorylated CBL and an activator of XIAP. Our work demonstrates the versatility of the UbV technology and provides a resource for the rapid development of inhibitors and activators across the large RING/U-box E3 family.

RESULTS

Identification of Selective UbVs for RING and U-box E3s

Using phage-displayed UbV library 2 (Zhang et al., 2016; Figure 1A) and a well-established strategy (Figure 1B), we conducted binding selections with the U-box from UBE4B (E4B, residues 1079–C) and the RING domains from Tyr371-phosphorylated c-CBL (pCBLR, residues 354–435) and XIAP (XR, residues 434–C). Aiming to isolate the highest-affinity variants, we increased stringency by reducing the target protein amount and increasing the washes in each selection round. After five rounds, phage ELISAs with 96 individual clones yielded 6, 90, and 77 positive clones for E4B, pCBLR, and XR, respectively. Consistent with the high stringency selection, DNA sequencing revealed a single unique UbV in each case (Figure 1A). Further phage ELISAs revealed that wild-type Ub did not bind or bound very weakly to nine RING/U-box E3s, whereas each selected UbV, named UbV.E4B, UbV.pCBL, and UbV.XR, bound with much higher affinity exclusively to E4B, pCBLR, and XR, respectively (Figure 1C). The specificities were validated by performing ELISAs with purified UbVs and Ub (Figure 1D).

To investigate the effects of the UbVs on ligase activity, we performed autoubiquitination assays with GST-tagged E4B, pCBLR, and XR. The E2 UbcH5B was charged with Ub before adding a mixture of each GST-tagged E3 domain with its cognate UbV or Ub lacking the C-terminal diglycine (Ub74) as a negative control. UbV.E4B and UbV.pCBL inhibited their cognate E3s, whereas, unexpectedly, UbV.XR stimulated autoubiquitination of GST-XR (Figure 1E). We next undertook further biochemical and structural studies of the three E3-UbV pairs to elucidate the molecular basis for selectivity and activity.

UbV.E4B Inhibits UBE4B via the E2~Ub Binding Site

UbV.E4B selectively inhibited E4B, the U-box domain of UBE4B (Figure 1). To elucidate the inhibitory mechanism, we measured the affinity of UbV.E4B for E4B and tested its activity in single-turnover lysine discharge assays with UbcH5B containing an S22R substitution (UbcH5B S22R). This activity assay was chosen to eliminate potential effects on acceptor Ub interactions in the enzyme-substrate complex and to prevent stimulation of Ub transfer from E2~Ub by non-covalent binding of Ub to UbcH5B's backside (Brzovic et al., 2006; Buetow et al., 2015). UbV.E4B bound to E4B with an affinity of 1.9 μM by surface plasmon resonance (SPR) (Table 1; Figure S1), but it only weakly inhibited E4B-mediated lysine discharge (Figure 2A), even at 500 μM.

UBE4B is known as a Ub elongation factor or E4 because it binds preformed Ub conjugates and catalyzes formation of polyubiquitin chains (Kaneko et al., 2003; Koegl et al., 1999). In cells, UBE4B only facilitates Ub chain elongation on p53 after initial ubiquitination by MDM2 (Wu et al., 2011). We speculated that UbV.E4B may have stronger effects on polyubiquitin chain formation and thus performed single-turnover Ub transfer autoubiquitination assays with GST-E4B. Indeed, formation of ubiquitinated GST-E4B was inhibited in a concentration-dependent manner by UbV.E4B at 100 or 500 μM, but there were no obvious effects on product formation by 20 μM UbV.E4B, despite being at approximately ten times the K_D (Figure 2B).

We next examined whether UbV.E4B functions in cells. Co-immunoprecipitation assays on lysates from HEK293T cells, in which hemagglutinin (HA)-tagged UbV.E4B (HA-UbV.E4B) and Myc-tagged UBE4B (Myc-UBE4B) were overexpressed, confirmed an interaction between UBE4B and UbV.E4B (Figure 2C). Moreover, HA-UbV.E4B, but not HA-Ub74, decreased ubiquitination of GFP-tagged p53 (Figure 2D). These data show that UbV.E4B binds UBE4B and inhibits its activity in cells.

We attempted to co-crystallize UbV.E4B with several E4B U-box variants of differing lengths. A crystal structure of UBE4B U-box comprising residue 1,097 to the C terminus ($E4B_{1097-C}$) was determined to a resolution of 1.48 Å (Table 2; Figure S2A), but no crystals of a complex with UbV.E4B were obtained. We therefore undertook nuclear magnetic resonance (NMR) experiments to map the complex interface. Since UBE4B primarily functions as an E4 ligase, E4B might

Figure 1. Identification of Selective UbVs for RING and U-box Domains

(A) Sequence alignment of Ub and UbVs selective for the RING domain of pCBL or XIAP or the U-box domain of UBE4B. The alignment only shows regions that were diversified in the UbV library. Dashes indicate positions in which the Ub sequence is conserved.

(B) The four-step procedures to conduct phage display selections for UbV binders of RING/U-box E3 ligases. Please refer to the STAR Methods for details of the selection cycle.

(C) The binding specificities of phage-displayed UbVs (y axis) are shown across a group of RING domains from nine E3s (x axis), as assessed by phage ELISA. Sub-saturating concentrations of UbV phage were added to immobilized proteins as indicated. Bound phage were detected by the addition of anti-M13-HRP and colorimetric development of TMB peroxidase substrate. The mean value of absorbance at 450 nm is shaded in a white-purple gradient.

(D) ELISAs for UbVs or Ub binding to RING/U-box E3s. GST-tagged RING/U-box domains from nine E3s (1 μM, 30 μL) were incubated with indicated amounts of FLAG-tagged UbV or Ub (0–62.5 nM, 30 μL). Bound UbV was detected by anti-FLAG-HRP conjugate antibody and colorimetric development of TMB peroxidase substrate. Absorbance at 450 nm (y axis) was plotted against UbV concentration (x axis). Data are presented as the mean ± SD (n = 3).

(E) Reduced autoradiograms showing the formation of ^{32}P-Ub products over time with GST-E4B (top), GST-pCBLR (center), or GST-XR (bottom) in the presence of Ub74 (left) or their respective UbVs (right).

Table 1. Dissociation Constants or Interactions between RING E3 Variants, UbVs, and UbcH5B S22R C85K-Ub

Immobilized Protein	Analyte[a]	K_D (μM)	Binding enhancement (Fold)[b]
E4B	UbV.E4B	1.9 ± 0.5	–
$E4B_{1097–C}$	UbV.E4B	9 ± 1	–
E4B L1107R	UbV.E4B	No binding	–
E4B T1122R	UbV.E4B	19 ± 3	–
E4B F1141R	UbV.E4B	25 ± 8	–
E4B R1143A	UbV.E4B	16 ± 2	–
pCBLR	UbV.pCBL	0.12 ± 0.02	–
CBLR	UbV.pCBL	No binding	–
$pCBL_{47-435}$	UbV.pCBL	0.25 ± 0.04	–
pCBLR M374R	UbV.pCBL	1.6 ± 0.2	–
pCBLR I383R	UbV.pCBL	Poor or no binding	–
pCBL-B	UbV.pCBL	1.3 ± 0.1	–
pCBL-C	UbV.pCBL	1.1 ± 0.2	–
XR	$UbV.XR_M$	1.1 ± 0.2	–
XR	$UbV.XR_D$	0.11 ± 0.02	–
XR	UbV.XR A10G	No binding	–
XR	UbV.XR K48F	2.4 ± 0.2	–
XR	UbV.XR K48R	0.21 ± 0.04	–
XR	UbcH5B S22R C85K–Ub	12.2 ± 0.3	–
XR	UbcH5B S22R C85K-Ub + 10 μM UbV.XR	5.5 ± 0.7	2.2
XR	UbcH5B S22R C85K-Ub + 10 μM UbV.XR K48F	15.1 ± 0.4	0.8
XR	UbcH5B S22R C85K-Ub + 10 μM UbV.XR K48R	4.0 ± 0.2	3.0

SEM and dissociation constants (K_D) are indicated. Number of replicates, representative sensorgrams, and binding curves are shown in Figure S1.
[a]Analytes containing fixed concentration of UbV.XR variants are indicated.
[b]The degree of binding enhancement of UbcH5B S22R C85K-Ub in the presence of UbV.XR is determined by dividing the K_D in the absence by the K_D in the presence of indicated UbV.XR variants.

preferentially interact with mono-Ub or polyUb chains. To rule this out, we titrated ^{15}N-Ub with E4B or K48-diUb with ^{15}N-E4B. In each case, negligible chemical shift perturbations (CSPs) were observed, suggesting that E4B does not directly interact with mono- or polyUb (Figures S2B and S2C).

With UbV.E4B in place of Ub, a dramatically different picture emerged. With ^{15}N-UbV.E4B, numerous large CSPs were observed across a number of peaks, including in one of the two tryptophan indole groups (Figure S3A), whereas in the titration of UbV.E4B into ^{15}N-E4B, the CSPs were more localized (Figure S3B). Residue-specific CSPs for ^{15}N-E4B were generated from these data (Figure 2E), and residues with CSPs > 1σ were mapped onto the structure of UBE4B in complex with UbcH5C (PDB: 3L1Z; Figure 2F). Next, we used SPR to investigate effects of substitutions at selected positions (L1107R, T1122R, F1141R, and R1143A) on UbV.E4B binding. Binding was either abrogated or reduced by 10- to 20-fold (Table 1; Figure S1). Notably, these CSPs on E4B mapped to the same residues involved in E2 and E2~Ub binding based on the crystal structure of the UBE4B-UbcH5C complex (Benirschke et al., 2010) (Figure 2F) and NMR chemical shift analysis of the UBE4B-UbcH5C~Ub complex (Pruneda et al., 2012), respectively. To investigate whether UbV.E4B and E2 compete for the same binding site on E4B, we monitored CSPs in ^{15}N-UbcH5B competition experiments. Addition of equimolar E4B to ^{15}N-UbcH5B strongly affected several residue peaks within the spectra indicating formation of ^{15}N-UbcH5B-E4B complex. Subsequent titration of UbV.E4B caused ^{15}N-UbcH5B signals to shift back to free E2 positions (Figure 2G; Figure S3C), showing that UbV.E4B inhibits E4B by occupying the E2-binding site.

Inhibition by UbV.pCBL Relies on Tyr371-Phosphorylation of CBL

The three human isoforms of CBL (c-CBL or CBL, CBL-B, and CBL-C) share homology between their N-terminal regions comprising a substrate tyrosine kinase binding domain (TKBD), linker, and RING domain (Swaminathan and Tsygankov, 2006). In cells, tyrosine kinase substrate ubiquitination by CBL requires phosphorylation of the conserved Tyr371, which resides on the α helix within the linker (Dou et al., 2012a; Levkowitz et al., 1999). To investigate the selectivity of UbV.pCBL, we measured its affinity for several CBL variants by SPR and tested its activity against these variants in single-turnover lysine discharge assays with UbcH5B S22R.

In native CBL, Tyr371 is buried in a pocket on the TKBD and stabilizes the RING domain in a catalytically incompetent conformation (Dou et al., 2012a; Zheng et al., 2000). Tyr371 phosphorylation abolishes the TKBD-linker interaction and frees the RING domain to adopt conformations in which the TKBD substrate-binding site is accessible. In addition, phosphorylated Tyr371 (pTyr371) locks into the RING domain and interacts with E2~Ub to prime it for catalysis (Dou et al., 2012a, 2013). Both unphosphorylated c-CBL RING (CBLR) and pCBLR were included in our panel of E3s, but only pCBLR bound to UbV.pCBL (Figure 1). Correspondingly, UbV.pCBL bound pCBLR tightly in SPR (K_D = 120 nM) but did not bind detectably to CBLR (Table 1; Figure S1). In activity assays, UbV.pCBL inhibited discharge of UbcH5B S22R~Ub by pCBLR, but not CBLR (Figure 3A). Because the RING-TKBD domain interactions change upon phosphorylation of Tyr371 (Dou et al., 2012a), we investigated whether this impacts RING-UbV interactions by conducting assays with a longer pCBL variant encompassing the TKBD, linker, and RING domains ($pCBL_{47–435}$). $pCBL_{47–435}$ and pCBLR had similar affinities for UbV.pCBL in SPR (K_D = 250 and 120 nM, respectively, Table 1; Figure S1), and formation of ubiquitinated-$pCBL_{47–435}$ and discharge of UbcH5B S22R~Ub in single-turnover Ub transfer assays were slowed by UbV.pCBL (Figure 3B). Together, these data show that UbV.pCBL binds pCBLR, but not CBLR, and inhibits RING-mediated Ub transfer.

We determined the crystal structure of UbV.pCBL in complex with $pCBL_{47–435}$ and the ZAP70-TKBD-substrate peptide to

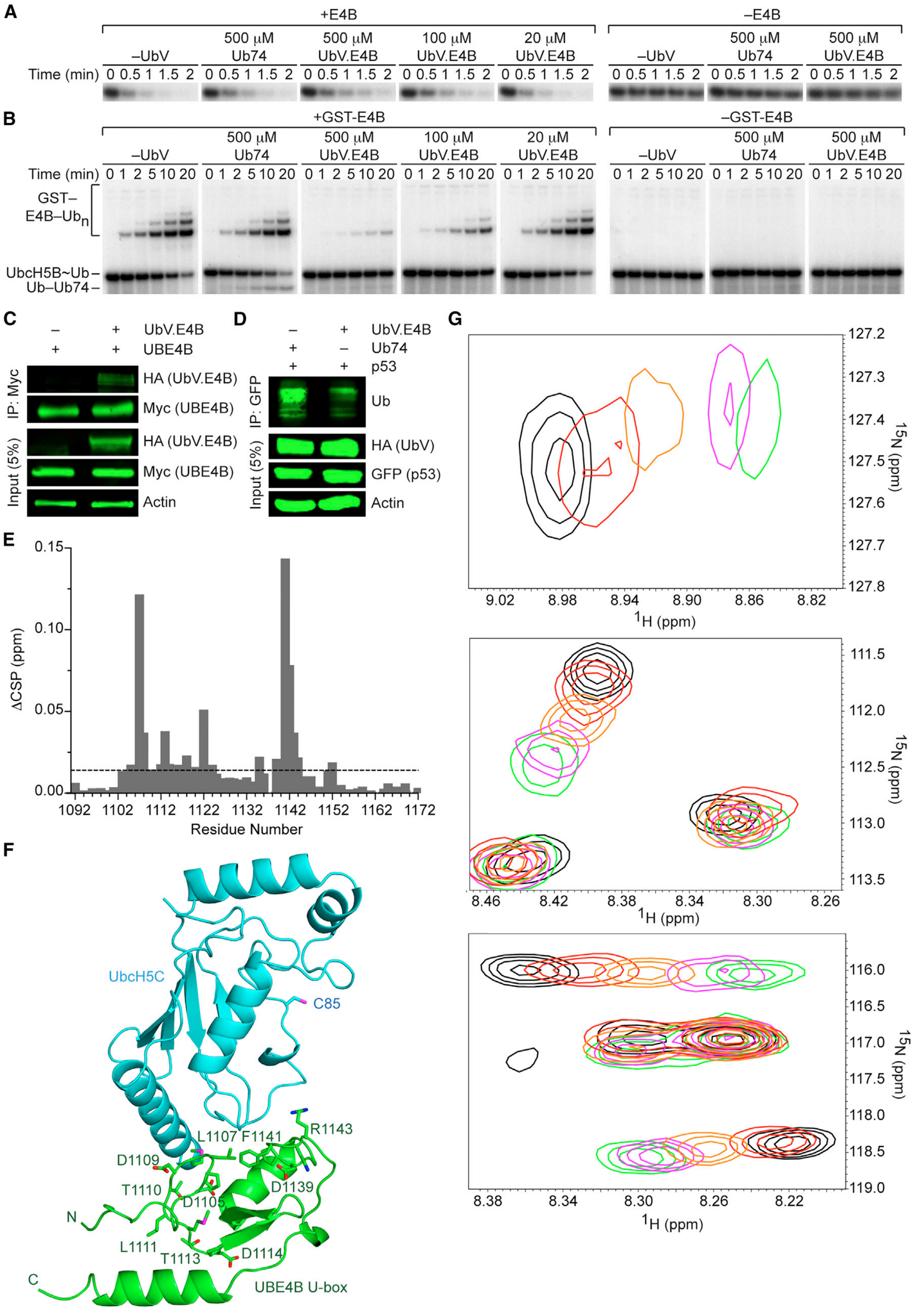

(legend on next page)

Table 2. Data Collection and Refinement Statistics

	XR-UbV.XR	UbV.XR	ZAP70 peptide-pCBL$_{47–435}$-UbV.pCBL	E4B$_{1097–C}$
Data Collection				
PDB	PDB: 5O6T	PDB: 5O6S	PDB: 5O76	PDB: 5O75
Space group	$P2_12_12_1$	$I2$	$P2_12_12_1$	$P622$
Cell dimensions				
a, b, c (Å)	35.7, 70.0, 109.8	48.2, 71.7, 118.0	94.8, 101.3, 117.3	80.2, 80.2, 39.5
α, β, γ (°)	90, 90, 90	90, 94.7, 90	90, 90, 90	90, 90, 120
Resolution (Å)	43–1.57 (1.61–1.57)[a]	61–2.90 (3.07–2.90)	35–2.47 (2.54–2.47)	40–1.48 (1.51–1.48)
R_{merge} (%)	6.6 (80.3)	16.9 (52.3)	11.2 (115.0)	4.3 (119.4)
R_{pim} (%)	4.2 (51.3)	16.7 (51.3)	6.6 (66.7)	1.5 (43.5)
Completeness (%)	100 (96.3)	99.4 (99.3)	93.3 (99.0)	91.9 (100.0)
Multiplicity	6.4 (5.0)	2.9 (2.9)	6.5 (6.9)	17.0 (15.2)
I/σI	14.9 (2.0)	3.7 (1.5)	13.9 (2.1)	27.9 (2.2)
CC(1/2)	0.999 (0.663)	0.966 (0.725)	0.998 (0.796)	1.000 (0.771)
Wilson B (Å^2)	20.57	58.40	59.34	25.70
Refinement				
R_{work} (%)	15.8	26.3	21.8	19.5
R_{free} (%)	18.6	30.9	24.8	22.4
No. atoms				
Protein	4961	3582	9266	549
Water	218	32	235	44
Ligand / ion	4	0	6	0
RMSD bond	0.01	0.008	0.008	0.008
RMSD angle	1.15	1.06	0.95	1.03
B factors				
Main chain	23.41	52.71	66.07	37.86
Side chain	30.92	64.57	72.96	43.63
Zn^{2+}	16.80	–	51.00	–
Water	40.87	20.37	54.88	48.15

[a]Values in parentheses are for highest resolution shell.

2.47 Å (Figure 3C; Table 2). In the complex, pCBL$_{47–435}$ is in the active conformation in which pTyr371 supports the E2~Ub binding site and the RING domain is in a catalytically competent conformation oriented toward the bound ZAP70 substrate peptide on the TKBD (Dou et al., 2012a, 2013). UbV.pCBL interacts with the E2~Ub binding site on the RING domain and

Figure 2. Mechanism of E4B Inhibition by UbV.E4B

(A) Non-reduced autoradiograms of single-turnover lysine discharge reactions showing the disappearance of UbcH5B S22R~^{32}P-Ub over time with lysine only or indicated concentrations of UbV.E4B or Ub74 in the presence (left) or absence (right) of E4B.

(B) Non-reduced autoradiograms of single-turnover GST-E4B mediated Ub transfer reactions showing the disappearance of UbcH5B S22R~^{32}P-Ub and appearance of ^{32}P-Ub$_n$-GST-E4B and other ^{32}P-Ub$_n$-products over time with indicated concentrations of UbV.E4B or Ub74 in the presence (left) or absence (right) of GST-E4B.

(C) Co-immunoprecipitation assay of lysates from HEK293T cells transfected with plasmids expressing Myc-tagged UBE4B and HA-tagged UbV.E4B. Immunoprecipitates with anti-Myc antibody and cell lysates were analyzed by immunoblotting with anti-Myc, anti-HA, or anti-Actin antibodies as indicated.

(D) Immunoblots of p53 ubiquitination in HEK293T cells transfected with plasmids expressing GFP-tagged p53 and HA-tagged UbV.pCBL or Ub74. The cell lysates and GFP immunoprecipitates were analyzed by immunoblotting with anti-Ub, anti-GFP, anti-HA, or anti-Actin antibodies as indicated.

(E) Changes in CSP (ΔCSP) determined by ^{1}H-^{15}N HSQC NMR for each residue of ^{15}N-E4B following addition of 1.77-fold molar excess of UbV.E4B. Changes were calculated according to the equation $[(\delta_{HA} - \delta_{HB})^2 + ((\delta_{NA} - \delta_{NB})/5)^2]^{1/2}$. The dashed line represents a ΔCSP value of 1σ (0.014 ppm), where σ corresponds to standard deviation. See also Figure S3B.

(F) Mapping of changes in CSPs from (E) (>1σ) onto the U-box domain of UBE4B in the structure of UBE4B (green) bound to UbcH5C (cyan) (PDB: 3L1Z). Residues that undergo perturbations are labeled and shown as sticks. The active site Cys85 on UbcH5C is also shown as sticks and labeled. N, O, and S atoms are colored blue, red, and magenta, respectively.

(G) CSP data of representative residue peaks from UbcH5B in competition with UbV.E4B for binding to E4B. ^{1}H-^{15}N HSQC spectra for free ^{15}N-UbcH5B (black), bound to E4B (green), and subsequently titrated with UbV.E4B where [UbV.E4B]:[^{15}N-UbcH5B] is 1:1 (magenta), 5:1 (orange), and 10:1 (red).

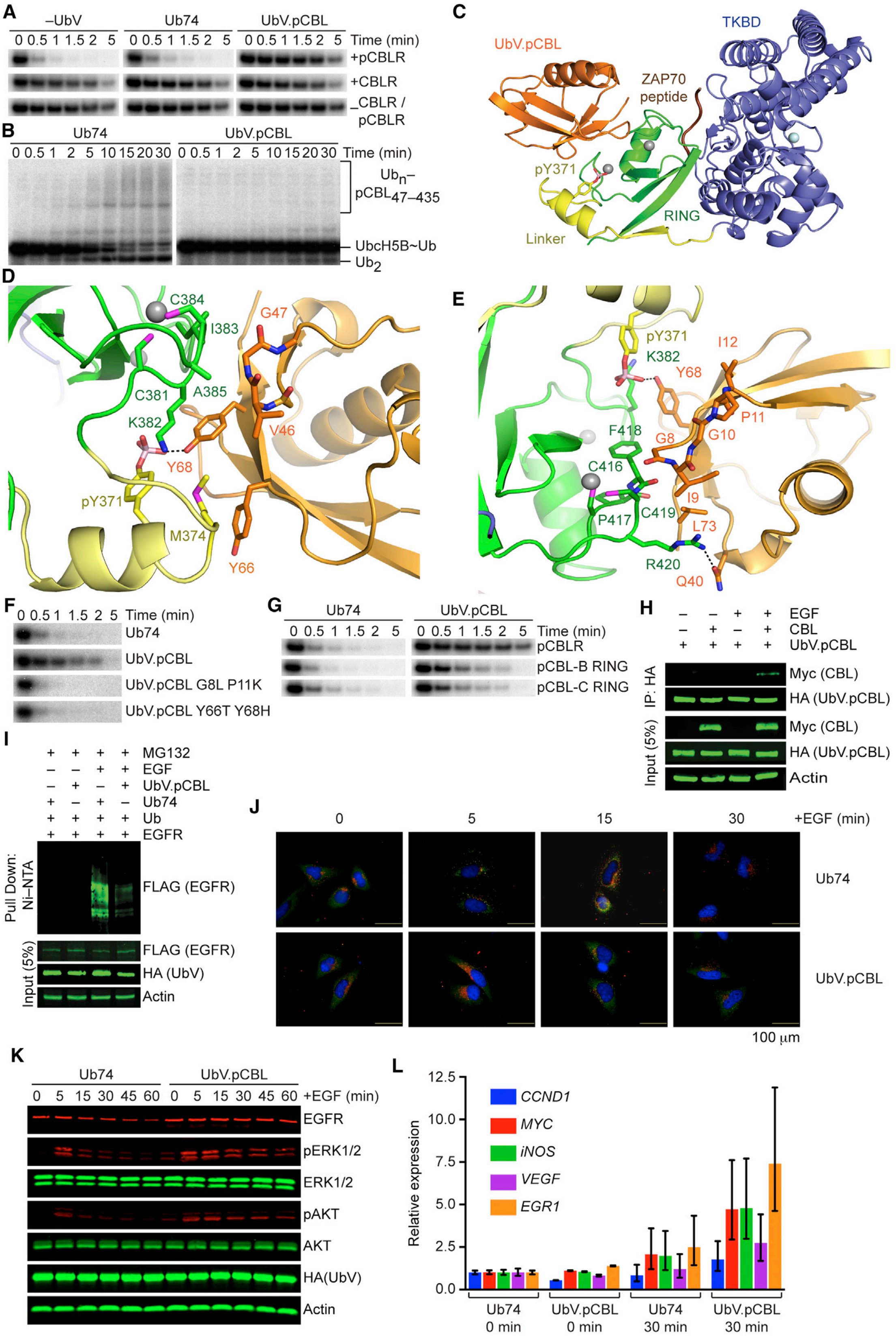

(legend on next page)

inhibits Ub transfer by blocking E2∼Ub binding (Figure 3C). Our $pCBL_{47–435}$ structure and a previous model (PDB: 4A4B) superpose with a root-mean-square-deviation (RMSD) of 1.37 Å for 379 Cα atoms, whereas the TKBD and linker/RING domains can be superposed with the corresponding regions with RMSD values of 1.21 and 0.64 Å for 303 Cα and 74 Cα atoms, respectively (Figures S4A–S4E).

The main difference between the two structures of $pCBL_{47–435}$ is a shift of the RING domain in relation to the TKBD caused by binding of UbV.pCBL (Figures S4A–S4E). UbV.pCBL resembles Ub, as the two superpose with an RMSD of 0.76 Å, but substitutions in region 3 (Figure 1) of the UbV mediate interactions with pTyr371; Tyr66 and Tyr68, replacing Thr66 and His68 in Ub, form a hydrophobic cap around Met374 at the C terminus of the linker helix and Tyr68 interacts with the phosphate moiety of pTyr371 (Figure 3D). UbV.pCBL also stacks against the second Zn^{2+}-binding site on the RING domain via a hydrophobic patch formed by substitutions in positions 8–12 (Figure 3E). To validate these interactions, we measured binding of UbV.pCBL to pCBLR variants containing an M374R or an I383R substitution, which are expected to perturb UbV interactions with the linker or the second Zn^{2+}-binding site, respectively. Binding was reduced in both cases, with a K_D of 1.6 μM for the M374R substituted variant and no detectable binding for the I383R substituted variant (Table 1; Figure S1). To investigate the roles of substitutions within regions 1 and 3 in conferring selectivity of UbV.pCBL, we reverted key positions in pairs to wild-type sequence (G8L/P11K and Y66T/Y68H). Both reversion variants failed to inhibit pCBLR-mediated discharge of UbcH5B S22R∼Ub (Figure 3F).

Next, we investigated the selectivity of UbV.pCBL for the RING domains from different CBL isoforms. CBLR shares 95% and 60% sequence identity with the RING domains of CBL-B and CBL-C, respectively. In SPR assays, UbV.pCBL bound pCBL-B and pCBL-C with K_Ds of 1.3 and 1.1 μM, respectively (Table 1; Figure S1) and inhibited discharge of UbcH5B S22R∼Ub by both E3 isoforms (Figure 3G). These data show that UbV.pCBL binds selectively and inhibits the activity of the three phosphorylated isoforms but does not recognize CBL in its unphosphorylated state.

In unstimulated cells, CBL is predominantly unphosphorylated and inactive. Stimulation with EGF induces phosphorylation of Tyr371, which activates CBL-mediated ubiquitination of EGFR (Dou et al., 2012a; Levkowitz et al., 1999). To determine whether UbV.pCBL can selectively bind pCBL in cells, we performed co-immunoprecipitation assays in HEK293T cells with overexpressed HA-tagged UbV.pCBL (HA-UbV.pCBL) and Myc-tagged CBL (Myc-CBL) with and without EGF stimulation. Immunoprecipitation of HA-UbV.pCBL only co-precipitated Myc-CBL following EGF stimulation, demonstrating that UbV.pCBL only binds CBL following an event that increases the population of pCBL (Figure 3H). Moreover, ubiquitination of EGFR only occurred following EGF stimulation but was decreased when UbV.pCBL was present, showing that UbV.pCBL inhibits ubiquitination of EGFR by pCBL (Figure 3I).

Endosomal trafficking and lysosomal degradation of EGFR are highly dependent on its ubiquitination (Tomas et al., 2014), so we examined whether UbV.pCBL could alter the fate of activated EGFR. UbV.pCBL decreased EGFR accumulation in early endosomes as evidenced by reduced co-localization of EGFR with the early endosomal marker EEA1 upon EGF stimulation (Figure 3J; Figure S4F). Activated EGFR was also more stable in the presence of UbV.pCBL, which resulted in prolonged downstream signaling events as shown by extended ERK1/2

Figure 3. Mechanism of pCBL Inhibition by UbV.pCBL

(A) Non-reduced autoradiograms of single-turnover lysine discharge reactions showing the disappearance of UbcH5B S22R∼^{32}P-Ub over time with pCBLR (top row), CBLR (middle row), or no E3 (bottom row) in the presence of lysine only (left), Ub74 (middle), or UbV.pCBL (right).

(B) Non-reduced autoradiograms of single-turnover Ub transfer reactions mediated by $pCBL_{47–435}$ showing the disappearance of UbcH5B S22R∼^{32}P-Ub and appearance of ^{32}P-Ub_n-$pCBL_{47–435}$ and other ^{32}P-Ub_n products over time in the presence of 10 μM Ub74 (left) or UbV.pCBL (right).

(C) Cartoon representation of the UbV.pCBL-$pCBL_{47–435}$ -ZAP70 substrate peptide complex. ZAP70 substrate peptide is colored brown, UbV.pCBL is colored orange, and the TKBD, linker, and RING domains of $pCBL_{47–435}$ are colored blue-gray, yellow, and green, respectively. Zn^{2+} and Ca^{2+} ions are depicted as gray and light cyan spheres, respectively. The side chain of pTyr371 is shown as sticks with oxygen and phosphorous atoms colored red and pink, respectively.

(D) Close-up view of the interface between UbV.pCBL and the region around pTyr371 of $pCBL_{47–435}$. Key interacting residues are shown as sticks. Coloring is as described in (C) and Figure 2. S and N atoms are colored magenta and blue, respectively. The dashed black line depicts a putative hydrogen bond.

(E) Close-up view of the interface between UbV.pCBL and the second Zn^{2+}-binding loop in $pCBL_{47–435}$. Coloring is as described in (D).

(F) Non-reduced autoradiograms of single-turnover lysine discharge reactions showing the disappearance of UbcH5B S22R∼^{32}P-Ub over time with pCBLR and UbV.pCBL variants as indicated.

(G) Non-reduced autoradiograms of single-turnover lysine discharge reactions showing the disappearance of UbcH5B S22R∼^{32}P-Ub over time with pCBLR (top row), pCBL-B (middle row), or pCBL-C (bottom row) and Ub74 (left) and UbV.pCBL (right).

(H) Co-immunoprecipitation assay of lysates from HEK293T cells transfected with plasmids expressing Myc-tagged CBL and HA-tagged UbV.pCBL with and without EGF stimulation. Immunoprecipitates with anti-HA antibody and cell lysates were analyzed by immunoblotting with anti-Myc, anti-HA, or anti-Actin antibodies as indicated.

(I) Immunoblots of EGFR ubiquitination with and without EGF stimulation from lysates of HEK293T cells transfected with plasmids expressing His-tagged Ub, FLAG-tagged EGFR, and HA-tagged UbV.pCBL or Ub74 and treated with MG132. The cell lysates and Ni-NTA pull-down products were analyzed by immunoblotting with anti-FLAG, anti-HA, or anti-Actin antibodies as indicated.

(J) Merged images from HeLa cells overexpressing UbV.pCBL or Ub74 and treated with EGF as indicated. Cells were incubated with anti-EGFR and anti-EEA1 primary antibodies, followed by secondary antibodies conjugated to AF488 (EGFR, green) or AF594 (EEA1, red). DAPI was used to stain the nuclei. Scale bars in each panel represent 100 μm.

(K) Immunoblots of cell lysates from HEK293T cells overexpressing HA-tagged UbV.pCBL or Ub74 and treated with EGF for indicated time. Lysates were analyzed by immunoblotting with anti-EGFR, anti-pERK1/2 (T202/Y204), anti-ERK1/2, anti-pAKT (S473), anti-AKT, anti-HA, and anti-Actin antibodies.

(L) Bar graphs showing transcript levels of EGFR-regulated genes from H1299 cells overexpressing UbV.pCBL or Ub74 with or without EGF treatment. Quantitative RT-PCR was performed to check the levels of *CCND1*, *MYC*, *iNOS*, *VEGF*, and *EGR1*. The bars represent 95% confidence intervals for relative expression.

and AKT phosphorylation (Figure 3K). Activated EGFR is a transcriptional co-activator of several oncogenes, such as the Cyclin D1 gene *CCND1*, *MYC*, *iNOS*, *VEGF*, and *EGR1* (Brand et al., 2011). We found that the transcript levels of these EGFR-regulated genes were increased in cells overexpressing UbV.pCBL after EGF stimulation (Figure 3L). Together, these data show that UbV.pCBL selectively binds and inhibits pCBL in cells, thereby perturbing the signaling and transcriptional activities of its substrate EGFR.

Dimeric UbV.XR Stimulates XIAP

UbV.XR binds selectively to the RING domain of XIAP (residues 434–C, referred to as XR), but not the RING domain of BIRC2 (residues 555–C, referred to as B2R), and stimulates autoubiquitination of XR (Figure 1). To validate these findings, we performed single-turnover lysine discharge assays using UbV.XR with XR or B2R. A multi-step purification of UbV.XR resulted in the isolation of two distinct fractions by gel filtration chromatography (Figure 4A). The earlier and later fractions eluted at volumes consistent with a dimer (UbV.XR_D) and a monomer (UbV.XR_M), respectively. At 10 μM, both fractions stimulated XR-mediated UbcH5B S22R~Ub discharge to a similar extent, whereas Ub74 did not (Figure 4B). Titration of the two UbV fractions revealed that UbV.XR_D stimulated XR slightly better at lower concentrations (Figures 4C and 4D). Correspondingly, UbV.XR_D bound XR with 10-fold higher affinity compared to UbV.XR_M in SPR assays (Table 1; Figure S1).

Due to its higher activity, we used UbV.XR_D for further studies. UbV.XR_D stimulated discharge of UbcH5B S22R~Ub by XR, but not by B2R (Figure 4E), and no interaction between B2R and UbV.XR_D was detected by SPR (Table 1; Figure S1). Furthermore, SPR did not detect any interaction between UbV.XR_D and the RING domain of BIRC3 or BIRC7 despite high sequence and structural homology between these proteins and XIAP (Table 1; Figure S1). Investigation of the stimulatory capabilities of UbV.XR_D in the context of autoubiquitination of full-length XIAP and ubiquitination of the XIAP substrate mature SMAC (mSMAC, residues 56–C) in single-turnover Ub transfer assays revealed that UbV.XR_D promoted Ub transfer in both assays (Figure 4F).

To examine UbV.XR activity in cells, we monitored ubiquitination of mSMAC by endogenous XIAP in HEK293T cells in which UbV.XR was overexpressed. We confirmed that the UbV and its cognate E3 interact in cells by performing pull-down experiments on lysates in which HA-tagged UbV.XR (HA-UbV.XR) and Myc-tagged XIAP (Myc-XIAP) were overexpressed (Figure 4G). Cells were exposed to etoposide to initiate mitochondrial outer membrane permeabilization and the release of mSMAC from the mitochondria. Upon induction of apoptosis, an increase of ubiquitinated mSMAC was detected in cells overexpressing UbV.XR (Figure 4H). These results show that UbV.XR is selective for XIAP and enhances its ligase activity in cells.

We determined the crystal structure of XR bound to UbV.XR_D to 1.57 Å (Figure 5A; Table 2) but were unable to obtain crystals of an XR-UbV.XR_M complex. XR adopts a dimeric RING domain arrangement similar to structures of BIRC3 and BIRC7 in which the C-terminal tail of each subunit interacts with the second subunit (Dou et al., 2012b; Mace et al., 2008). UbV.XR_D forms a symmetrical domain-swapped dimer in which β1 is flipped 180° away from the β sheet of its own subunit and is replaced by $\beta1'$ from the other subunit. Whereas residues 6–9 in wild-type Ub form the loop between β1 and β2, in the UbV dimer they extend β1 and participate in hydrogen bonding with $\beta2'$ and $\beta1'$ to form a continuous anti-parallel β sheet across the two subunits (Figure 5A). We also determined the crystal structure of UbV.XR_D alone to 2.9 Å resolution and observed a dimer conformation similar to that of the dimer in the complex with XR, but the relative orientations of the two subunits differed by 30° (RMSD of 0.46 Å for 66 Cα atoms of a single subunit of UbV.XR_D and RMSD of 3.3 Å for 133 Cα atoms of both subunits of UbV.XR_D, Figures S5A–S5C; Table 2), suggesting conformational flexibility between the two UbV.XR subunits when not in complex. Apart from the domain swap, the overall fold of a single subunit of UbV.XR_D is similar to that of wild-type Ub, with an RMSD of 0.4 Å for 72 Cα atoms, if $\beta1'$ is treated as β1 (Figure S5D).

In the complex, the UbV dimer forms a groove that cradles the RING dimer (Figure 5A), covering a surface area of 816 $Å^2$ that is remote from the E2~Ub binding site. The groove is formed by the $\beta2$-$\beta1'$-$\beta1$-$\beta2'$ β sheet that comprises the UbV dimer interface. Two symmetrical hydrophobic pockets at the base of the groove bind the two Phe490 residues from the XR dimer (Figure 5B). Residues from both subunits of UbV.XR_D comprise each hydrophobic pocket and include substitutions relative to wild-type Ub (Figures 1 and 5B). Symmetrical salt bridges between UbV.XR_D Glu46 side chains (Ala46 in Ub) and XR Lys472 side chains also appear to stabilize the complex.

To validate these interactions, we tested the binding and activity of variants that contained substitutions in residues from the complex interface: T489E or F490R substitution in XR and I8R or L68R substitution in UbV.XR (Figure 5B). Both XR variants had elution volumes comparable to wild-type XR (data not shown) and UbV.XR L68R had an elution volume comparable to UbV.XR_D, whereas UbV.XR I8R had an elution volume comparable to UbV.XR_M (Figure 5C). UbV.XR_D did not stimulate UbcH5B S22R~Ub discharge catalyzed by XR T489E or XR F490R, even though both variants were comparably active to wild-type XR in promoting UbcH5B S22R~Ub discharge in the presence of Ub74 (Figure 4E). Similarly, neither UbV.XR I8R nor UbV.XR L68R stimulated UbcH5B S22R~Ub discharge catalyzed by XR (Figure 5D).

Modeling suggests that if the UbV.XR monomer adopts a fold similar to wild-type Ub, it cannot form the continuous β sheet comprising the XR-binding groove. Yet, UbV.XR_M still retains the ability to bind XR and stimulate UbcH5B S22R~Ub discharge (Figure 4B; Figures S1, S5E, and S5F; Table 1). To better understand this phenomenon, we used gel filtration chromatography to monitor the stability of UbV.XR monomer and dimer over time and found that the monomer was slowly converted to dimer whereas the dimer was stable (there was no evidence of dissociation to monomer or formation of larger oligomers (Figure 4A). We sought to substitute a residue that would abrogate formation of the UbV dimer but would not affect interactions with XR, but all residues that form the UbV dimer interface also interact with XR. We postulated that reverting Ala10 to Gly as found in Ub might fulfill our criteria because only the main chain of Ala10 is involved in interactions with XR, whereas the side

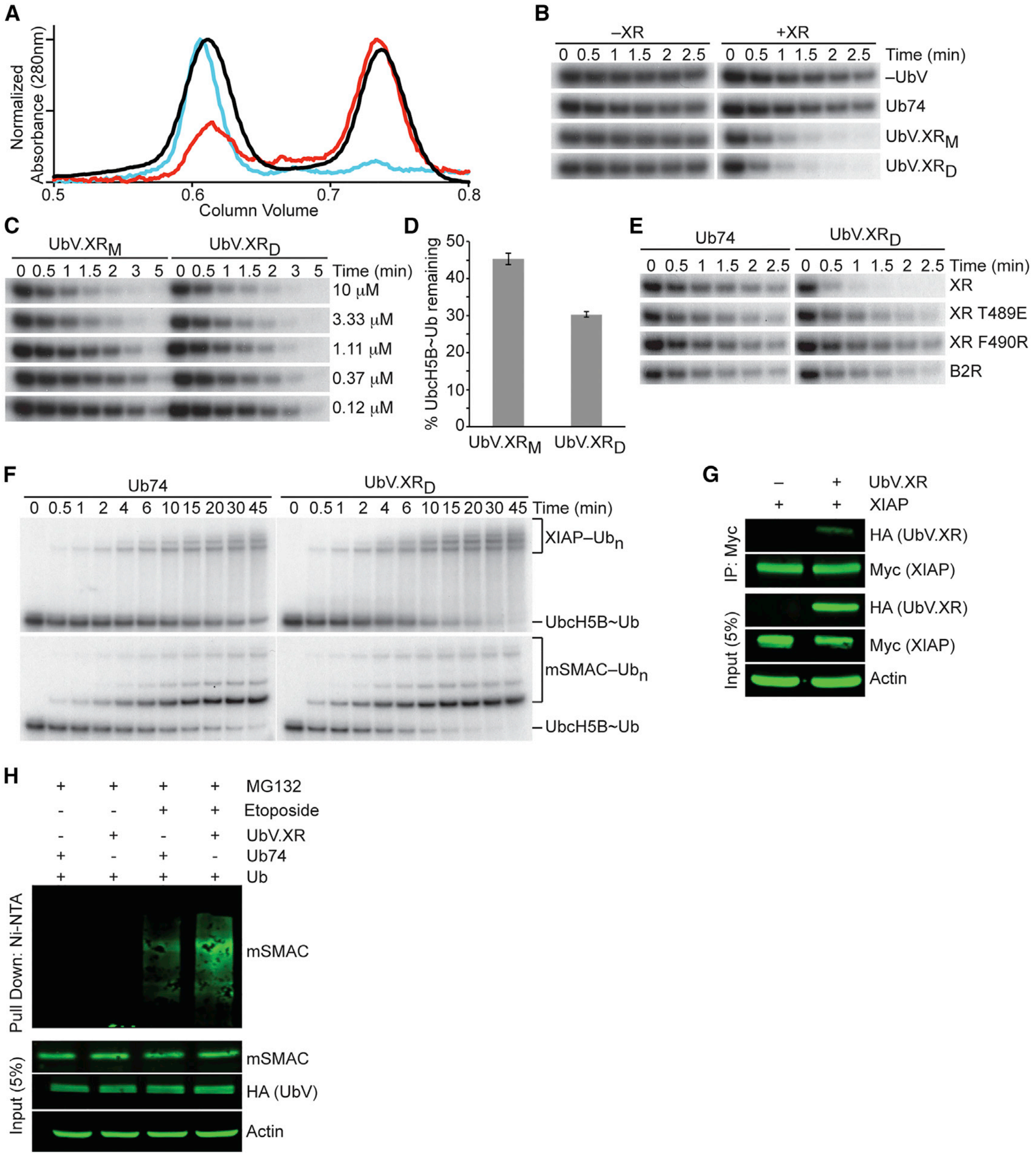

Figure 4. UbV.XR Stimulates the E3 Ligase Activity of XIAP *In Vitro* and In Cells

(A) Scaled Superdex75 chromatograms of UbV.XR during purification (black) showing the protein eluting at volumes consistent with dimer (UbV.XR_D) and monomer (UbV.XR_M), of UbV.XR_D after 7 days at 4°C (cyan), and of UbV.XR_M after 7 days at 4°C (red).

(B) Non-reduced autoradiograms of single-turnover lysine discharge reactions showing the disappearance of UbcH5B S22R~^{32}P-Ub over time in the absence (left) or presence (right) of XR with lysine only (top row), Ub74 (second row), UbV.XR_M (third row), or UbV.XR_D (fourth row).

(C) Non-reduced autoradiograms of single-turnover lysine discharge reactions showing the disappearance of UbcH5B S22R~^{32}P-Ub over time with XR in the presence of indicated concentrations of UbV.XR_M or UbV.XR_D. Concentrations were determined from A_{280} measurements using the calculated molar extinction coefficient and predicted mass of a monomer.

(D) Quantification of single-turnover lysine discharge as shown in (C) at 1.5 min with 1.11 μM UbV.XR_M or UbV.XR_D. Data are presented as an average ± 1σ (n = 4).

(E) Non-reduced autoradiograms of single-turnover lysine discharge reactions showing the disappearance of UbcH5B S22R~^{32}P-Ub over time with XR variants or B2R in the presence of Ub74 (left) or UbV.XR_D (right).

(F) Non-reduced autoradiograms of XIAP mediated single-turnover Ub transfer reactions showing the disappearance of UbcH5B S22R~^{32}P-Ub and appearance of ^{32}P-Ub_n-XIAP (top) or ^{32}P-Ub_n-mSMAC (bottom) over time in the presence of Ub74 (left) or UbV.XR_D (right).

(G) Co-immunoprecipitation assay of lysates from HEK293T cells transfected with plasmids expressing Myc-tagged XIAP and HA-tagged UbV.XR. Immunoprecipitates with anti-Myc antibody and cell lysates were analyzed by immunoblotting with anti-Myc, anti-HA, or anti-Actin antibodies as indicated.

(H) Immunoblots of HEK293T cell lysates to detect ubiquitination of mSMAC by XIAP in the presence of UbV.XR or Ub74, with or without etoposide and treated with MG132. Ni-NTA pull-down products/cell lysates were analyzed by immunoblotting with anti-SMAC, anti-HA, and anti-Actin antibodies as indicated.

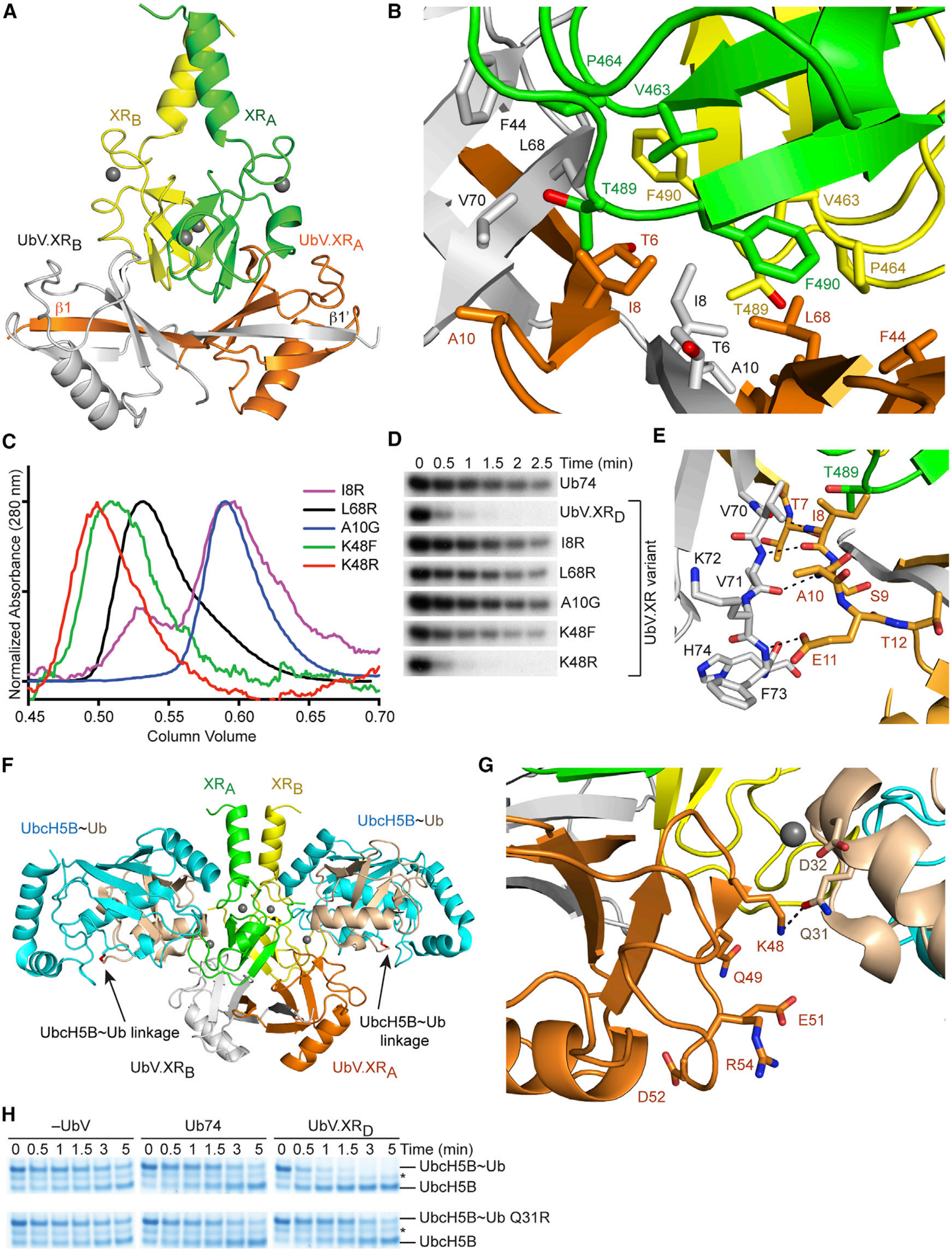

Figure 5. Proposed Mechanism for the Activation of XIAP by UbV.XR

(A) Cartoon representation of XR in complex with UbV.XR$_D$. The subunits of the XR dimer are colored green and yellow and those of UbV.XR$_D$ are colored orange and white. Zn^{2+} ions are depicted as gray spheres.

(B) Close-up view of the UbV.XR$_D$-XR dimer interface. Key residues are shown as sticks and coloring is as in (A) and Figure 3.

(C) Overlaid gel filtration Superdex 75 chromatograms of UbV.XR I8R (purple), UbV.XR L68R (black), UbV.XR A10G (blue), UbV.XR K48F (green), and UbV.XR K48R (red).

(legend continued on next page)

chain is buried in a pocket on the second UbV subunit (Figure 5E). Gel filtration chromatography revealed that UbV.XR A10G was exclusively a monomer during purification and remained a monomer after incubation at 4°C for 1 week (Figure 5C; data not shown). The UbV.XR A10G variant showed no evidence of binding to XR in SPR assays (Table 1; Figure S1) and did not stimulate XR-mediated UbcH5B S22R~Ub discharge (Figure 5D). Taken together, these findings suggest that UbV.XR dimerization is critical for XR binding and stimulation.

To elucidate how UbV.XR stimulates Ub transfer by XR, we generated a model of the XR-UbV.XR_D complex bound to UbcH5B~Ub based on the structure of the BIRC7-UbcH5B-Ub complex (Dou et al., 2012b) (Figure 5F). The model suggests that UbV.XR_D contacts the C terminus of α1 from donor Ub independently of its interactions with XR. We postulated that these interactions might help position donor Ub for transfer. To test this hypothesis, we generated stably conjugated UbcH5B S22R C85K-Ub to mimic UbcH5B~Ub as described (Dou et al., 2013), and SPR analyses of XR binding to this stable conjugate showed that UbV.XR_D enhanced affinity by 2-fold (Table 1; Figure S1).

An electrostatic surface potential map of our model suggests that interactions between UbV.XR and donor Ub may involve an acidic patch surrounding Asp32 on Ub and a basic patch encompassing the region between Lys48 and Arg54 on UbV.XR (Figures S6A–S6C). One of these electrostatic interactions is a putative hydrogen bond between Lys48 of UbV.XR_D and Gln31 of Ub (Figure 5G). We postulated that these electrostatic interactions might help prime donor Ub for transfer, so we substituted Gln31 on Ub with Arg and examined whether UbV.XR_D could promote XR-mediated discharge of UbcH5B S22R~Ub Q31R. In the presence of Ub74, discharge of UbcH5B S22R~Ub and UbcH5B S22R~Ub Q31R by XR were comparable whereas with UbV.XR_D, discharge of the former E2~Ub was stimulated, but not the latter (Figure 5H). Next, we substituted Lys48 of UbV.XR_D with Phe or Arg and tested the ability of these variant dimers to stimulate XR in discharge assays. We predicted that a Phe48 substitution would disrupt the electrostatic interactions whereas an Arg48 substitution would not. UbV.XR K48F did not stimulate XR-mediated discharge of UbcH5B S22R~Ub whereas UbV.XR K48R did (Figure 5D). SPR analyses of XR binding to UbcH5B S22R C85K-Ub in the presence of each of these UbVs showed that, like UbV.XR_D, UbV.XR K48R enhanced binding by 3-fold whereas UbV.XR K48F did not (Table 1; Figure S1). These results support our hypothesis that when bound to XR, UbV.XR_D interacts directly with donor Ub to help stabilize it in a primed conformation, thereby stimulating XR-mediated Ub transfer.

DISCUSSION

There is a paucity of technologies to selectively modulate components of the ubiquitination pathway at the protein level in cells. RING and U-box E3s pose a particular challenge for probe development because they often have multiple substrate-binding domains, as in the case of XIAP, or they have functions beyond Ub ligation, as in the case of CBL. Moreover, some RING and U-box domains exhibit very high sequence identity, thus making probe selectivity problematic. Here, we used a phage-displayed library to identify UbVs that selectively bound and modulated the activity of UBE4B, pCBL or XIAP. UbV.E4B and UbV.pCBL occupy the E2~Ub binding sites of their respective U-box and RING domains and inhibit Ub transfer, whereas UbV.XR forms a dimer that occupies a site on the RING dimer in which it stabilizes donor Ub and stimulates Ub transfer.

Our UbV library was originally designed to develop inhibitors of deubiquitinases (Ernst et al., 2013). Recently, we showed that UbVs could exhibit multiple binding modes and mechanisms to modulate HECT E3 activity (Zhang et al., 2016)—one set occupied the HECT domain E2-binding site and inhibited Ub ligation. Here, our structural studies show that the E2~Ub-binding site of RING/U-box is also targetable by UbVs. Therefore, a generalizable strategy is now available to potentially generate potent and specific inhibitors for over 300 human RING/U-box domains.

Our study of HECT E3s also identified UbVs that bind to a Ub-binding exosite and stimulate ubiquitination activity (Zhang et al., 2016). In contrast to HECT domains, in which a large surface area of ~19,000 $Å^2$ is available for binding, RING and U-box domains only present a total surface area of ~5,000 $Å^2$ or ~7,600 $Å^2$ as monomers or dimers, respectively. Given this relatively small surface area, it is noteworthy that UbV.XR activates E3 activity through binding to a region on XIAP that has not previously been reported to be involved in protein-protein interactions. These data demonstrate the versatility of our UbV technology for exploiting both canonical and non-canonical protein-protein interaction surfaces of RING and U-box domains to develop selective probes.

All three UbVs were active *in vitro* and in cells, thereby providing valuable tools for probing the cellular functions of UBE4B, CBL, and XIAP. In addition to regulating p53 stability and function (Wu et al., 2011), ubiquitination by UBE4B also plays a role in endosomal sorting and lysosomal degradation of EGFR and probably regulates sorting of other membrane proteins (Sirisaengtaksin et al., 2014). UbV.E4B will be useful for discovering additional pathways subject to UBE4B-mediated ubiquitination. CBL functions as both an adaptor and a negative regulator in tyrosine kinase-mediated signaling. It attenuates

(D) Non-reduced autoradiograms of single-turnover lysine discharge reactions showing the disappearance of UbcH5B S22R~^{32}P-Ub over time with XR in the presence of Ub74 or UbV.XR variants as indicated.

(E) Close-up view of UbV.XR_D Ala10. Residues within 5 Å are shown as sticks and coloring is as in (A) and Figure 3.

(F) Model of UbV.XR-XR complex bound to UbcH5B-Ub. The model was generated by superposing the RING domains from BIRC7 RING-UbcH5B-Ub complex (PDB: 4AUQ) and UbV.XR-XR without any adjustment to residue positions. Coloring is as described in (A); UbcH5B is colored cyan and donor Ub wheat. An arrow points to the UbcH5B-Ub linkage (in red).

(G) Close-up of interactions between UbV.XR and donor Ub. Coloring is as described in (E) and (F).

(H) SDS-PAGE of single-turnover lysine discharge reactions showing the disappearance of UbcH5B S22R~Ub (top) or UbcH5B S22R~Ub Q31R (bottom) over time with XR in the presence of lysine only (left), Ub74 (middle), or UbV.XR_D (right).

signaling by ubiquitinating receptor and non-receptor tyrosine kinases and targeting them for degradation. Substrate ubiquitination involves the N-terminal region of CBL encompassing the TKBD, linker, and RING domain and requires phosphorylation of Tyr371, whereas the adaptor functions of CBL are mediated by a proline-rich region and the C terminus (Levkowitz et al., 1999; Swaminathan and Tsygankov, 2006). UbV.pCBL only binds CBL when it is phosphorylated on Tyr371, thus providing a tool to differentiate between its ligase and adaptor functions in cells. XIAP inhibits apoptosis by binding Caspase-3, -7, and -9, and it activates NFκB signaling by binding the upstream adaptor TAB1. Moreover, it is upregulated in several cancers and confers resistance to chemotherapy-induced cell death (Obexer and Ausserlechner, 2014). Small molecule inhibitors, such as SMAC mimetics, have been developed to target the baculovirus-IAP-repeat (BIR) domain, but these molecules also target the BIR domains of the inhibitor of apoptosis family E3s (Bai et al., 2014). The E3 ligase activity of the RING domain of XIAP controls its stability and ubiquitinates substrates such as Caspase-3 and mSMAC (MacFarlane et al., 2002; Suzuki et al., 2001). The specificity of UbV.XR for XIAP and its role as a stimulator of E3 activity will enable further studies of hyperactive XIAP in cells and will enhance understanding of how E3 activity could influence its function.

Our data suggest that UbV.XR stimulates XIAP-mediated Ub transfer by binding the RING domain and stabilizing donor Ub in a conformation primed for catalysis. Only one other RING E3 has a similar mechanism: free Ub binds the RING domain of the monomeric E3 Arkadia and is postulated to directly contact and position donor Ub for transfer (Wright et al., 2016). However, UbV.XR and free Ub bind to different regions of the XIAP and Arkadia RING domains, respectively, and consequently contact different surfaces on donor Ub. Moreover, our data indicate that UbV.XR must dimerize to stimulate XIAP-mediated Ub transfer.

The β strand domain swap in the UbV.XR dimer appears to be a unique arrangement for a Ub-based dimer. In solution, free Ub has a K_D of ~5 mM for the monomer-dimer equilibrium, and the two subunits in the dimer adopt a range of relative orientations involving residues from Ub's β sheet (Liu et al., 2012). Once UbV.XR dimer has formed, no shift to monomer is observed (Figure 4A), but the two dimeric subunits have some degree of flexibility relative to one another. Comparison to a range of Ub dimers involving isopeptide linkages with the N terminus or one of Ub's Lys residues reveals that UbV.XR most closely resembles compact diUb chains linked through Lys6, Lys11, or Lys33 (RMSD ranging from 5.4 to 6.2 Å) (Bremm et al., 2010; Hospenthal et al., 2013; Matsumoto et al., 2010; Virdee et al., 2010), but modeling XR with any of these diUb chains superposed onto UbV.XR reveals potential clashes. We identified the G10A substitution in UbV.XR as being essential for UbV dimerization, but other substitutions may also contribute. Importantly, a phage-displayed dimeric UbV library can now be constructed using UbV.XR as a template, and such a library holds great potential for developing activators of other IAP proteins and other dimeric RING/U-box E3s.

A powerful aspect of phage display technology is that libraries can be improved in response to new insights gained from the results of selection experiments, functional analyses, and structural information. Although Ub interacts with most of its binding partners through a similar interface, there are significant differences in terms of the residues that make contacts with different structural folds. Thus, structural analysis of the interaction interfaces for UbVs generated thus far will allow us to more accurately define the type of diversity that should be included at each position within the UbV binding surface. These insights can be used to design further optimized libraries that are likely to yield more potent UbVs for targeting particular structural folds.

In summary, we took advantage of the UbV technology to identify modulators of RING and U-box E3 ligases, which can be used in cells to explore ubiquitination pathways and signaling. In addition to identifying competitive inhibitors that target the E2~Ub binding sites of RING and U-box E3s, we identified a distinct surface on XIAP RING dimer that stimulated E3 ligase activity when bound to dimeric UbV.XR. Remarkably, UbV.pCBL recognized a specific active conformation of CBL induced by phosphorylation of Tyr371. We conclude that the UbV technology provides a unified platform for the rapid development of both inhibitors and activators of the large RING/U-box E3 family.

STAR★METHODS

Detailed methods are provided in the online version of this paper and include the following:

- KEY RESOURCES TABLE
- CONTACT FOR REAGENT AND RESOURCE SHARING
- EXPERIMENTAL MODEL AND SUBJECT DETAILS
  - Recombinant Proteins
  - Cell Culture and Transfection
- METHOD DETAILS
  - Selection of Ubiquitin Variants
  - ELISAs to Evaluate Binding and Specificity
  - Generation of Constructs
  - Protein Purification
  - SPR Binding Assays
  - Solution NMR Experiments
  - Analytical Gel Filtration
  - Crystallization
  - Data Collection and Structure Determination
  - Autoubiquitination Assays
  - Single-Turnover Lysine Discharge Assays
  - Single-Turnover Ub Transfer Reactions
  - Chemicals and Antibodies for Cell Culture
  - Co-immunoprecipitation and Immunoblotting
  - Ubiquitination Assays in Cells
  - Immunocytochemistry
  - cDNA Synthesis and Quantitative Real-Time PCR
- QUANTIFICIATION AND STATISTICAL ANALYSIS
- DATA AND SOFTWARE AVAILABILITY

SUPPLEMENTAL INFORMATION

Supplemental Information includes six figures and can be found with this article online at https://doi.org/10.1016/j.molcel.2017.09.027.

AUTHOR CONTRIBUTIONS

Conceptualization, M.G., L.B., W.Z., S.S.S., and D.T.H.; Methodology, M.G., L.B., W.Z., M.A.N., S.F.A., B.O.S., S.S.S., and D.T.H.; Investigation, M.G., L.B., W.Z., M.A.N., B.O.S., S.F.A., and G.J.S.; Writing – Original Draft, M.G. and L.B.; Writing – Review & Editing, L.B., M.G., W.Z., M.A.N., D.T.H., and S.S.S.; Supervision, S.S.S. and D.T.H.; Funding Acquisition, S.S.S. and D.T.H.

ACKNOWLEDGMENTS

We thank W. Clark and A. Keith for in-house DNA sequencing, J. Gu for technical assistance in phage display, and Diamond Light Source for access to stations I03 and I04 (BAG allocation mx11651). W.Z. is supported by the Mitacs Elevate program. This work was supported by Cancer Research UK (C596/A23278) and European Research Council (ERC) under the European Union's Horizon 2020 research and innovation programme (grant agreement n° 647849) awarded to D.T.H. and a Genome Canada Disruptive Innovation in Genomics grant (OGI-119) and a CQDM-OCE EXPLORE Project (#23927) awarded to S.S.S.

Received: June 15, 2017
Revised: August 9, 2017
Accepted: September 20, 2017
Published: October 19, 2017

REFERENCES

Adams, P.D., Afonine, P.V., Bunkóczi, G., Chen, V.B., Davis, I.W., Echols, N., Headd, J.J., Hung, L.W., Kapral, G.J., Grosse-Kunstleve, R.W., et al. (2010). PHENIX: a comprehensive Python-based system for macromolecular structure solution. Acta Crystallogr. D Biol. Crystallogr. *66*, 213–221.

Ahmed, S.F., Das, N., Sarkar, M., Chatterjee, U., Chatterjee, S., and Ghosh, M.K. (2015). Exosome-mediated delivery of the intrinsic C-terminus domain of PTEN protects it from proteasomal degradation and ablates tumorigenesis. Mol. Ther. *23*, 255–269.

Bai, L., Smith, D.C., and Wang, S. (2014). Small-molecule SMAC mimetics as new cancer therapeutics. Pharmacol. Ther. *144*, 82–95.

Benirschke, R.C., Thompson, J.R., Nominé, Y., Wasielewski, E., Juranić, N., Macura, S., Hatakeyama, S., Nakayama, K.I., Botuyan, M.V., and Mer, G. (2010). Molecular basis for the association of human E4B U box ubiquitin ligase with E2-conjugating enzymes UbcH5c and Ubc4. Structure *18*, 955–965.

Brand, T.M., Iida, M., Li, C., and Wheeler, D.L. (2011). The nuclear epidermal growth factor receptor signaling network and its role in cancer. Discov. Med. *12*, 419–432.

Bremm, A., Freund, S.M., and Komander, D. (2010). Lys11-linked ubiquitin chains adopt compact conformations and are preferentially hydrolyzed by the deubiquitinase Cezanne. Nat. Struct. Mol. Biol. *17*, 939–947.

Bricogne, G., Blanc, E., Brandl, M., Flensburg, C., Keller, P., Paciorek, W., Roversi, P., Sharff, A., Smart, O.S., Vonrhein, C., et al. (2016). BUSTER version X.Y.Z (Global Phasing Ltd.).

Brown, N.G., VanderLinden, R., Watson, E.R., Weissmann, F., Ordureau, A., Wu, K.P., Zhang, W., Yu, S., Mercredi, P.Y., Harrison, J.S., et al. (2016). Dual RING E3 architectures regulate multiubiquitination and ubiquitin chain elongation by APC/C. Cell *165*, 1440–1453.

Brzovic, P.S., Lissounov, A., Christensen, D.E., Hoyt, D.W., and Klevit, R.E. (2006). A UbcH5/ubiquitin noncovalent complex is required for processive BRCA1-directed ubiquitination. Mol. Cell *21*, 873–880.

Buetow, L., and Huang, D.T. (2016). Structural insights into the catalysis and regulation of E3 ubiquitin ligases. Nat. Rev. Mol. Cell Biol. *17*, 626–642.

Buetow, L., Gabrielsen, M., Anthony, N.G., Dou, H., Patel, A., Aitkenhead, H., Sibbet, G.J., Smith, B.O., and Huang, D.T. (2015). Activation of a primed RING E3-E2-ubiquitin complex by non-covalent ubiquitin. Mol. Cell *58*, 297–310.

Bulatov, E., and Ciulli, A. (2015). Targeting Cullin-RING E3 ubiquitin ligases for drug discovery: structure, assembly and small-molecule modulation. Biochem. J. *467*, 365–386.

Cai, M., Huang, Y., Sakaguchi, K., Clore, G.M., Gronenborn, A.M., and Craigie, R. (1998). An efficient and cost-effective isotope labeling protocol for proteins expressed in Escherichia coli. J. Biomol. NMR *11*, 97–102.

Chen, V.B., Arendall, W.B., 3rd, Headd, J.J., Keedy, D.A., Immormino, R.M., Kapral, G.J., Murray, L.W., Richardson, J.S., and Richardson, D.C. (2010). MolProbity: all-atom structure validation for macromolecular crystallography. Acta Crystallogr. D Biol. Crystallogr. *66*, 12–21.

Deshaies, R.J., and Joazeiro, C.A. (2009). RING domain E3 ubiquitin ligases. Annu. Rev. Biochem. *78*, 399–434.

Dou, H., Buetow, L., Hock, A., Sibbet, G.J., Vousden, K.H., and Huang, D.T. (2012a). Structural basis for autoinhibition and phosphorylation-dependent activation of c-Cbl. Nat. Struct. Mol. Biol. *19*, 184–192.

Dou, H., Buetow, L., Sibbet, G.J., Cameron, K., and Huang, D.T. (2012b). BIRC7-E2 ubiquitin conjugate structure reveals the mechanism of ubiquitin transfer by a RING dimer. Nat. Struct. Mol. Biol. *19*, 876–883.

Dou, H., Buetow, L., Sibbet, G.J., Cameron, K., and Huang, D.T. (2013). Essentiality of a non-RING element in priming donor ubiquitin for catalysis by a monomeric E3. Nat. Struct. Mol. Biol. *20*, 982–986.

Emsley, P., Lohkamp, B., Scott, W.G., and Cowtan, K. (2010). Features and development of Coot. Acta Crystallogr. D Biol. Crystallogr. *66*, 486–501.

Ernst, A., Avvakumov, G., Tong, J., Fan, Y., Zhao, Y., Alberts, P., Persaud, A., Walker, J.R., Neculai, A.M., Neculai, D., et al. (2013). A strategy for modulation of enzymes in the ubiquitin system. Science *339*, 590–595.

Evans, P. (2006). Scaling and assessment of data quality. Acta Crystallogr. D Biol. Crystallogr. *62*, 72–82.

Evans, P.R., and Murshudov, G.N. (2013). How good are my data and what is the resolution? Acta Crystallogr. D Biol. Crystallogr. *69*, 1204–1214.

Gasteiger, E., Hoogland, C., Gattiker, A., Duvaud, S., Wilkins, M.R., Appel, R.D., and Bairoch, A. (2005). Protein identification and analysis tools on the ExPASy Server. In The proteomics protocols handbook, J.M. Walker, ed. (Humana Press), pp. 571–607.

Gorelik, M., Orlicky, S., Sartori, M.A., Tang, X., Marcon, E., Kurinov, I., Greenblatt, J.F., Tyers, M., Moffat, J., Sicheri, F., and Sidhu, S.S. (2016). Inhibition of SCF ubiquitin ligases by engineered ubiquitin variants that target the Cul1 binding site on the Skp1-F-box interface. Proc. Natl. Acad. Sci. USA *113*, 3527–3532.

Goru, S.K., Pandey, A., and Gaikwad, A.B. (2016). E3 ubiquitin ligases as novel targets for inflammatory diseases. Pharmacol. Res. *106*, 1–9.

Hershko, A., and Ciechanover, A. (1998). The ubiquitin system. Annu. Rev. Biochem. *67*, 425–479.

Hospenthal, M.K., Freund, S.M., and Komander, D. (2013). Assembly, analysis and architecture of atypical ubiquitin chains. Nat. Struct. Mol. Biol. *20*, 555–565.

Huang, D.T., Zhuang, M., Ayrault, O., and Schulman, B.A. (2008). Identification of conjugation specificity determinants unmasks vestigial preference for ubiquitin within the NEDD8 E2. Nat. Struct. Mol. Biol. *15*, 280–287.

Kabsch, W. (2010). Xds. Acta Crystallogr. D Biol. Crystallogr. *66*, 125–132.

Kaneko, C., Hatakeyama, S., Matsumoto, M., Yada, M., Nakayama, K., and Nakayama, K.I. (2003). Characterization of the mouse gene for the U-box-type ubiquitin ligase UFD2a. Biochem. Biophys. Res. Commun. *300*, 297–304.

Kleywegt, G.J. (1996). Use of non-crystallographic symmetry in protein structure refinement. Acta Crystallogr. D Biol. Crystallogr. *52*, 842–857.

Koegl, M., Hoppe, T., Schlenker, S., Ulrich, H.D., Mayer, T.U., and Jentsch, S. (1999). A novel ubiquitination factor, E4, is involved in multiubiquitin chain assembly. Cell *96*, 635–644.

Komander, D., and Rape, M. (2012). The ubiquitin code. Annu. Rev. Biochem. *81*, 203–229.

Krissinel, E., and Henrick, K. (2007). Inference of macromolecular assemblies from crystalline state. J. Mol. Biol. *372*, 774–797.

Landré, V., Rotblat, B., Melino, S., Bernassola, F., and Melino, G. (2014). Screening for E3-ubiquitin ligase inhibitors: challenges and opportunities. Oncotarget *5*, 7988–8013.

Levkowitz, G., Waterman, H., Ettenberg, S.A., Katz, M., Tsygankov, A.Y., Alroy, I., Lavi, S., Iwai, K., Reiss, Y., Ciechanover, A., et al. (1999). Ubiquitin ligase activity and tyrosine phosphorylation underlie suppression of growth factor signaling by c-Cbl/Sli-1. Mol. Cell *4*, 1029–1040.

Liu, Z., Zhang, W.P., Xing, Q., Ren, X., Liu, M., and Tang, C. (2012). Noncovalent dimerization of ubiquitin. Angew. Chem. Int. Ed. Engl. *51*, 469–472.

Mace, P.D., Linke, K., Feltham, R., Schumacher, F.R., Smith, C.A., Vaux, D.L., Silke, J., and Day, C.L. (2008). Structures of the cIAP2 RING domain reveal conformational changes associated with ubiquitin-conjugating enzyme (E2) recruitment. J. Biol. Chem. *283*, 31633–31640.

MacFarlane, M., Merrison, W., Bratton, S.B., and Cohen, G.M. (2002). Proteasome-mediated degradation of Smac during apoptosis: XIAP promotes Smac ubiquitination in vitro. J. Biol. Chem. *277*, 36611–36616.

Manczyk, N., Yates, B.P., Veggiani, G., Ernst, A., Sicheri, F., and Sidhu, S.S. (2017). Structural and functional characterization of a ubiquitin variant engineered for tight and specific binding to an α-helical ubiquitin interacting motif. Protein Sci. *26*, 1060–1069.

Matsumoto, M.L., Wickliffe, K.E., Dong, K.C., Yu, C., Bosanac, I., Bustos, D., Phu, L., Kirkpatrick, D.S., Hymowitz, S.G., Rape, M., et al. (2010). K11-linked polyubiquitination in cell cycle control revealed by a K11 linkage-specific antibody. Mol. Cell *39*, 477–484.

McCoy, A.J., Grosse-Kunstleve, R.W., Adams, P.D., Winn, M.D., Storoni, L.C., and Read, R.J. (2007). Phaser crystallographic software. J. Appl. Cryst. *40*, 658–674.

Nakatani, Y., Kleffmann, T., Linke, K., Condon, S.M., Hinds, M.G., and Day, C.L. (2013). Regulation of ubiquitin transfer by XIAP, a dimeric RING E3 ligase. Biochem. J. *450*, 629–638.

Nalepa, G., Rolfe, M., and Harper, J.W. (2006). Drug discovery in the ubiquitin-proteasome system. Nat. Rev. Drug Discov. *5*, 596–613.

Obexer, P., and Ausserlechner, M.J. (2014). X-linked inhibitor of apoptosis protein - a critical death resistance regulator and therapeutic target for personalized cancer therapy. Front. Oncol. *4*, 197.

Petroski, M.D. (2008). The ubiquitin system, disease, and drug discovery. BMC Biochem. *9* (*Suppl 1*), S7.

Pickart, C.M., and Eddins, M.J. (2004). Ubiquitin: structures, functions, mechanisms. Biochim. Biophys. Acta *1695*, 55–72.

Plechanovová, A., Jaffray, E.G., Tatham, M.H., Naismith, J.H., and Hay, R.T. (2012). Structure of a RING E3 ligase and ubiquitin-loaded E2 primed for catalysis. Nature *489*, 115–120.

Popovic, D., Vucic, D., and Dikic, I. (2014). Ubiquitination in disease pathogenesis and treatment. Nat. Med. *20*, 1242–1253.

Pruneda, J.N., Littlefield, P.J., Soss, S.E., Nordquist, K.A., Chazin, W.J., Brzovic, P.S., and Klevit, R.E. (2012). Structure of an E3:E2∼Ub complex reveals an allosteric mechanism shared among RING/U-box ligases. Mol. Cell *47*, 933–942.

Schindelin, J., Arganda-Carreras, I., Frise, E., Kaynig, V., Longair, M., Pietzsch, T., Preibisch, S., Rueden, C., Saalfeld, S., Schmid, B., et al. (2012). Fiji: an open-source platform for biological-image analysis. Nat. Methods *9*, 676–682.

Sirisaengtaksin, N., Gireud, M., Yan, Q., Kubota, Y., Meza, D., Waymire, J.C., Zage, P.E., and Bean, A.J. (2014). UBE4B protein couples ubiquitination and sorting machineries to enable epidermal growth factor receptor (EGFR) degradation. J. Biol. Chem. *289*, 3026–3039.

Suzuki, Y., Nakabayashi, Y., and Takahashi, R. (2001). Ubiquitin-protein ligase activity of X-linked inhibitor of apoptosis protein promotes proteasomal degradation of caspase-3 and enhances its anti-apoptotic effect in Fas-induced cell death. Proc. Natl. Acad. Sci. USA *98*, 8662–8667.

Swaminathan, G., and Tsygankov, A.Y. (2006). The Cbl family proteins: ring leaders in regulation of cell signaling. J. Cell. Physiol. *209*, 21–43.

Tomas, A., Futter, C.E., and Eden, E.R. (2014). EGF receptor trafficking: consequences for signaling and cancer. Trends Cell Biol. *24*, 26–34.

Tonikian, R., Zhang, Y., Boone, C., and Sidhu, S.S. (2007). Identifying specificity profiles for peptide recognition modules from phage-displayed peptide libraries. Nat. Protoc. *2*, 1368–1386.

Varadan, R., Walker, O., Pickart, C., and Fushman, D. (2002). Structural properties of polyubiquitin chains in solution. J. Mol. Biol. *324*, 637–647.

Virdee, S., Ye, Y., Nguyen, D.P., Komander, D., and Chin, J.W. (2010). Engineered diubiquitin synthesis reveals Lys29-isopeptide specificity of an OTU deubiquitinase. Nat. Chem. Biol. *6*, 750–757.

Volk, S., Wang, M., and Pickart, C.M. (2005). Chemical and genetic strategies for manipulating polyubiquitin chain structure. Methods Enzymol. *399*, 3–20.

Vonrhein, C., Flensburg, C., Keller, P., Sharff, A., Smart, O., Paciorek, W., Womack, T., and Bricogne, G. (2011). Data processing and analysis with the autoPROC toolbox. Acta Crystallogr. D Biol. Crystallogr. *67*, 293–302.

Winter, G. (2010). xia2: an expert system for macromolecular crystallography data reduction. J. Appl. Cryst. *43*, 186–190.

Wright, J.D., Mace, P.D., and Day, C.L. (2016). Secondary ubiquitin-RING docking enhances Arkadia and Ark2C E3 ligase activity. Nat. Struct. Mol. Biol. *23*, 45–52.

Wu, H., Pomeroy, S.L., Ferreira, M., Teider, N., Mariani, J., Nakayama, K.I., Hatakeyama, S., Tron, V.A., Saltibus, L.F., Spyracopoulos, L., and Leng, R.P. (2011). UBE4B promotes Hdm2-mediated degradation of the tumor suppressor p53. Nat. Med. *17*, 347–355.

Zhang, W., Wu, K.P., Sartori, M.A., Kamadurai, H.B., Ordureau, A., Jiang, C., Mercredi, P.Y., Murchie, R., Hu, J., Persaud, A., et al. (2016). System-wide modulation of HECT E3 ligases with selective ubiquitin variant probes. Mol. Cell *62*, 121–136.

Zhang, W., Bailey-Elkin, B.A., Knaap, R.C.M., Khare, B., Dalebout, T.J., Johnson, G.G., van Kasteren, P.B., McLeish, N.J., Gu, J., He, W., et al. (2017a). Potent and selective inhibition of pathogenic viruses by engineered ubiquitin variants. PLoS Pathog. *13*, e1006372.

Zhang, W., Sartori, M.A., Makhnevych, T., Federowicz, K.E., Dong, X., Liu, L., Nim, S., Dong, A., Yang, J., Li, Y., et al. (2017b). Generation and validation of intracellular ubiquitin variant Inhibitors for USP7 and USP10. J. Mol. Biol. Published online June 3, 2017. https://doi.org/10.1016/j.jmb.2017.05.025.

Zheng, N., Wang, P., Jeffrey, P.D., and Pavletich, N.P. (2000). Structure of a c-Cbl-UbcH7 complex: RING domain function in ubiquitin-protein ligases. Cell *102*, 533–539.

STAR★METHODS

KEY RESOURCES TABLE

REAGENT or RESOURCE	SOURCE	IDENTIFIER
Antibodies		
Monoclonal anti-M13-HRP conjugate	GE Healthcare	Cat#27942101; RRID: AB_2616587
Mouse monoclonal anti-FLAG M2-Peroxidase (HRP)	Sigma-Aldrich	Cat#A8592; RRID: AB_439702
Rabbit anti-FLAG	Sigma-Aldrich	Cat#F7425; RRID: AB_439687
Mouse monoclonal anti-HA	Merck Millipore	Cat#05-904; RRID: AB_11213751
Mouse monoclonal anti-Myc-tag	Cell Signaling Technology	Cat#2276; RRID: AB_331783
Goat polyclonal anti-actin (I-19)	Santa Cruz Biotechnology	Cat#Sc-1616; RRID: AB_630836
Mouse monoclonal anti-Smac/Diablo	Abcam	Cat#Ab111893; RRID: AB_10862924
Donkey anti-goat IgG, IRDye 800CW conjugated	LI-COR Biosciences	Cat#926-32214; RRID: AB_621846
Goat anti-mouse IgG, IRDye 800CW conjugated	LI-COR Biosciences	Cat#926-32210; RRID: AB_621842
Goat anti-rabbit IgG, IRDye 800CW conjugated	LI-COR Biosciences	Cat#926-32211; RRID: AB_621843
Goat anti-Rabbit IgG (H+L) Cross-Adsorbed Secondary Antibody, Alexa 488	Thermo Fisher Scientific	Cat# A-11008; RRID: AB_143165
Goat anti-Rabbit IgG (H+L) Cross-Adsorbed Secondary Antibody, Alexa 488	Thermo Fisher Scientific	Cat# A-11005; RRID: AB_2534073
Mouse monoclonal anti-EEA1	BD Transduction Laboratories	Cat#610457; RRID: AB_397830
Rabbit polyclonal anti-EGFR	Merck Millipore	Cat#06-847; RRID: AB_2096607
Rabbit polyclonal anti-phospho-Akt (Ser473)	Cell Signaling Technology	Cat#9271; RRID: AB_329825
Mouse monoclonal anti-Akt (pan)	Cell Signaling Technology	Cat#2920; RRID: AB_2273787
Rabbit polyclonal anti-phospho-p44/42-MAPK (Erk1/2) (Thr202/Tyr204)	Cell Signaling Technology	Cat#9101; RRID: AB_331646
Mouse monoclonal p44/42 MAPK (Erk1/2)	Cell Signaling Technology	Cat#4696; RRID: AB_390780
Rabbit polyclonal anti-ubiquitin	Merck Millipore	Cat#662099; RRID: AB_2238524
Mouse monoclonal anti-GFP	Cusabio Life Science	Cat#CSB-MA000051M0m; RRID: AB_2687558
VECTASHIELD Mounting Medium with DAPI antibody	Vector Laboratories	Cat# H-1200; RRID: AB_2336790
Chemicals, Peptides, and Recombinant Proteins		
2-mercaptoethanol 99% 14.3 M(BME)	Sigma-Aldrich	Cat#M3148
Adenosine 5′-triphosphate, disodium, trihydrate (ATP)	Fisher Scientific	Cat#10326943
Ammonium Chloride (^{15}N, 99%)	Cambridge Isotopes	Cat#NLM-467
Apyrase from potato [ATPase:ADPase 1:1]	Sigma-Aldrich	Cat#A6535
Albumin, from Bovine Serum	Sigma-Aldrich	Cat#A3294
cOmplete protease inhibitor cocktail	Roche	Cat#11836145001
Creatine phosphokinase from bovine heart	Sigma-Aldrich	Cat#C7886
Dithiothreitol (DTT)	Formedium	Cat#DTT025
Etoposide (4′-Demethylepipodophyllotoxin 9-(4,6-O-ethylidene-β-D-glucopyranoside)	Sigma-Aldrich	Cat#E1383
9.25 MBθ γ-^{32}P-ATP (6,000 Ci/mmol 10 mCi/mL EasyTide) in 50 mM Tricine (pH 7.6)	PerkinElmer	Cat#NEG502Z250UC

(*Continued on next page*)

Continued

REAGENT or RESOURCE	SOURCE	IDENTIFIER
Glutathione, reduced, free acid	Fisher Scientific UK	Cat#11483074
hEGF Epidermal Growth Factor, human recombinant expressed in *E. coli*, suitable for cell culture	Sigma-Aldrich	Cat#E9644
Imidazole BioUltra	Sigma-Aldrich	Cat#56749
Pyrophosphatase, Inorganic from baker's yeast	Sigma-Aldrich	Cat#I1643
Isopropyl-β-D-1-thiogalactoside (IPTG)	Formedium	Cat#IPTG025
L-lysine monohydrate	Formedium	Cat#DOC0160
MG132	Calbiochem Merck Millipore	Cat#474790
Phenylmethanesulfonyl fluoride (PMSF)	Sigma-Aldrich	Cat#P7626
Phosphocreatine disodium salt hydrate	Sigma-Aldrich	Cat#P7936
Sodium orthovanadate	Sigma-Aldrich	Cat#S6508
Triton X-100	Sigma-Aldrich	Cat#T8787
Formaldehyde solution	Sigma-Aldrich	Cat#252549
IGEPAL CA-630	Sigma-Aldrich	Cat#18896
GST-E4B, HisGST-E4B, E4B and variants (including $E4B_{1097–C}$)	Buetow et al., 2015; This paper	N/A
HisGST-RNF38 RING	Buetow et al., 2015; This paper	N/A
HisGST-XR, XR, and variants	Buetow et al., 2015; This paper	N/A
HisGST-B2R	This paper	N/A
HisGST-pCBLR, pCBLR, CBLR and variants	Dou et al., 2012a; This paper	N/A
GST-BRE1A RING	This paper	N/A
GST-RNF2/BMI1 RING	Dou et al., 2012b; This paper	N/A
GST-MDM2/MDM4 RING	Dou et al., 2012b; This paper	N/A
Arabadopsis thaliana Uba1	Dou et al., 2012b; Huang et al., 2008	N/A
UbcH5B	Dou et al., 2012a; This paper	N/A
^{32}P-Ub	Huang et al., 2008	N/A
K48-diUb	Varadan et al., 2002	N/A
UbV.pCBL and variants	This paper	N/A
UbV.E4B	This paper	N/A
UbV.XR and variants	This paper	N/A
Ub74	Volk et al., 2005; This paper	N/A
UbcH5B S22R	Dou et al., 2012b; This paper	N/A
HisGST-$pCBL_{47–435}$ and $pCBL_{47–435}$	Dou et al., 2012a; This paper	N/A
HisGST pCBL-B and pCBL-B (RING domain)	Dou et al., 2013; This paper	N/A
HisGST pCBL-C and pCBL-C (RING domain)	This paper	N/A
GST-BIRC3 RING	Dou et al., 2012b; This paper	N/A
GST-BIRC7 RING	Dou et al., 2012b; This paper	N/A
pGEX4T1 HG TEV XIAP	Buetow et al., 2015	N/A
mSMAC	Buetow et al., 2015	N/A
Ub Q31R	This paper	N/A
Critical Commercial Assays		
Classics suite	QIAGEN	Cat#130701
JCSG+	QIAGEN	Cat#130720
Morpheus screen	Molecular Dimensions	Cat#MD1-46

(*Continued on next page*)

Continued

REAGENT or RESOURCE	SOURCE	IDENTIFIER
DyNAmo HS SYBR green qPCR kit	Thermo Scientific	Cat#F-410L
High capacity cDNA reverse transcription kit	Thermo Scientific	Cat#4368814
Deposited Data		
XR-UbV.XR (PDB: 5O6T)	This paper	PDB: 5O6T
UbV.XR (PDB: 5O6S)	This paper	PDB: 5O6S
ZAP70 peptide-CBL$_{47–435}$-UbV.pCBL (PDB: 5O76)	This paper	PDB: 5O76
E4B$_{1097–C}$ (PDB: 5O75)	This paper	PDB: 5O75
Raw data and images	This paper and Mendeley Data	https://doi.org/10.17632/hxd3cyxzrc.1
Experimental Models: Cell Lines		
HEK293T	ATCC	Cat#CRL-3216; RRID: CVCL_0063
H1299	ATCC	Cat#CRL-5803; RRID: CVCL_0060
HeLa	DSMZ	Cat#ACC-57; RRID: CVCL_0030
Recombinant DNA		
pGEX4T1 HG CBLR Y368F 354–435	Dou et al., 2012a	N/A
pGEX4T1 TEV BRE1A 870–C	This paper	N/A
pGEX4T1 TEV RNF2 1–114	Dou et al., 2012b	N/A
pRSFDuet TEV BMI1 1–109	Dou et al., 2012b	N/A
pGEX4T1 TEV MDM2 428–C	Dou et al., 2012b	N/A
pRSFDuet TEV MDM4 428–C	Dou et al., 2012b	N/A
pET-23d *Arabadopsis* Uba1	Huang et al., 2008	N/A
pRSF1b UbcH5B	Dou et al., 2012a	N/A
pGEX-2TK TEV Ub	Huang et al., 2008	N/A
pGEX4T1 HG TEV GGS-UbV.pCBL	This paper	N/A
pET-HisSmt3 GGS-UbV.pCBL	This paper	N/A
pRSFDuet TEV GGS-UbV.pCBL	This paper	N/A
pGEX4T1 HG TEV GGS-UbV.E4B	This paper	N/A
pRSFDuet TEV GGS-UbV.E4B	This paper	N/A
pGEX4T1 HG TEV GGS-UbV.XR	This paper	N/A
pRSFDuet TEV GGS-UbV.XR	This paper	N/A
pET-3a Ub74	Volk et al., 2005	N/A
pRSFDuet TEV GGS-Ub74	This paper	N/A
pRSF1b UbcH5B S22R	Dou et al., 2012b	N/A
pGEX4T1 HG TEV E4B 1079–C L1107R	This paper	N/A
pGEX4T1 HG TEV E4B 1079–C T1122R	This paper	N/A
pGEX4T1 HG TEV E4B 1079–C F1141R	This paper	N/A
pGEX4T1 HG TEV E4B 1079–C R1143A	This paper	N/A
pGEX4T1 HG CBL Y368F 47–435	Dou et al., 2012a	N/A
pGEX4T1 HG CBL-B Y360F 346–427	Dou et al., 2013	N/A
pGEX4T1 HG CBL-C Y402F 324–405	This paper	N/A
pGEX4T1 HG TEV E4B$_{1097–C}$	This paper	N/A
pET-HisSmt3 E4B$_{1097–C}$	This paper	N/A
pGEX4T1 TEV BIRC3 541–C	Dou et al., 2012b	N/A
pGEX4T1 TEV BIRC7 239–C	Dou et al., 2012b	N/A
pGEX4T1 HG TEV XIAP	Buetow et al., 2015	N/A
pET-23d mSMAC-2TK 56–C	Buetow et al., 2015	N/A
pRSFDuet TEV GGS-Ub Q31R	This paper	N/A

(*Continued on next page*)

Continued

REAGENT or RESOURCE	SOURCE	IDENTIFIER
pGEX4T1 HG TEV XR 434–C T489E	This paper	N/A
pGEX4T1 HG TEV XR 434–C F490R	This paper	N/A
pET-HisSmt3 GGS-UbV.pCBL G8L P11K	This paper	N/A
pET-HisSmt3 GGS-UbV.pCBL Y66T Y68GH	This paper	N/A
pRSFDuet TEV GGS-UbV.XR I8R	This paper	N/A
pRSFDuet TEV GGS-UbV.XR L68R	This paper	N/A
pRSFDuet TEV GGS-UbV.XR A10G	This paper	N/A
pRSFDuet TEV GGS-UbV.XR K48F	This paper	N/A
pRSFDuet TEV GGS-UbV.XR K48R	This paper	N/A
pRK5 HA-Ub74	This paper	N/A
pRK5 HA-UbV.pCBL	This paper	N/A
pRK5 HA-UbV.XR	This paper	N/A
pcDNA3.1 Myc/His-CBL	This paper	N/A
pcDNA3.1 Myc/His-XIAP	This paper	N/A
pcDNA3.1 FLAG-EGFR	This paper	N/A
pcDNA3.1 His-Ub	Gift from A. Hock	Dou et al., 2012a
pEGFP p53	Addgene	Cat#12091
Oligonucleotides		
18S rRNA F	5′-GCTTAATTTGACTCAACACGGGC-3′	N/A
18S rRNA R	5′- AGCTATCAATCTGTCAATCCTGTC-3′	N/A
CCND1 F	5′-CCGTCCATGCGGAAGATC-3′	N/A
CCND1 R	5′- GAAGACCTCCTCCTCGCACT-3′	N/A
MYC F	5′-CCAACAGGAACTATGACCTCGACTAC-3′	N/A
MYC R	5′-CTCGAATTTCTTCCAGATATCCT-3′	N/A
VEGF F	5′- AAATGCTTTCTCCGCTCTGA −3′	N/A
VEGF R	5′- CCCACTGAGGAGTCCAACAT −3′	N/A
iNOS F	5′-CAGCGGGATGACTTTCCAA-3′	N/A
iNOS R	5′-AGGCAAGATTTGGACCTGCA-3′	N/A
EGR1 F	5′-TTCGGATCCTTTCCTCACTC-3′	N/A
EGR1 R	5′- GTTGCTCAGCAGCATCATCT-3′	N/A
Software and Algorithms		
GraphPad Prism	GraphPad Software	https://www.graphpad.com; RRID: SCR_002798
ProtParam	Gasteiger et al., 2005	http://web.expasy.org/protparam/; RRID: SCR_012880
Biacore T200 BIAevaluation	GE Healthcare	http://www.biacore.com; RRID: SCR_008424N/A
Scrubber2	BioLogic Software	http://www.biologic.com.au/scrubber.html; RRID: SCR_015745
xia2 pipeline	Winter, 2010	http://xia2.github.io; RRID: SCR_015746
XDS	Kabsch, 2010	http://xds.mpimf-heidelberg.mpg.de/; RRID: SCR_015652
POINTLESS	Evans, 2006	http://www.ccp4.ac.uk/html/pointless.html; RRID: SCR_014218
AIMLESS	Evans and Murshudov, 2013	http://www.ccp4.ac.uk/html/aimless.html; RRID: SCR_015747
autoPROC	Vonrhein et al., 2011	https://www.globalphasing.com/autoproc/; RRID: SCR_015748

(*Continued on next page*)

**Continued**		
REAGENT or RESOURCE	SOURCE	IDENTIFIER
PHASER	McCoy et al., 2007	http://www.ccp4.ac.uk/html/phaser.html; RRID: SCR_014219
PHENIX	Adams et al., 2010	https://www.phenix-online.org/; RRID: SCR_014224
BUSTER	Bricogne et al., 2016	https://www.globalphasing.com/buster/; RRID: SCR_015653
COOT	Emsley et al., 2010	https://www2.mrc-lmb.cam.ac.uk/personal/pemsley/coot/; RRID: SCR_014222
MOLPROBITY	Chen et al., 2010	http://molprobity.biochem.duke.edu/; RRID: SCR_014226
LSQMAN	Kleywegt, 1996	http://xray.bmc.uu.se/usf/lsqman_man.html; RRID: SCR_015751
PyMOL	The PyMOL Molecular Graphics System, v.1.8.4.0, Schrodinger, LLC	https://pymol.org/; RRID: SCR_000305
PISA	Krissinel and Henrick, 2007	http://www.ebi.ac.uk/pdbe/pisa/; RRID: SCR_015749
Top Spin v3.1	Bruker	https://www.bruker.com/products/mr/nmr/nmr-software/software/topspin/overview.html; RRID: SCR_014227
UCSF Sparky	N/A	http://www.cgl.ucsf.edu/home/sparky; RRID: SCR_014228
ImageQuantTL 8.1	GE Healthcare	http://www.gelifesciences.com/webapp/wcs/stores/servlet/catalog/en/GELifeSciences-us/products/AlternativeProductStructure_16016/29000605; RRID: SCR_014246N
Fiji	Schindelin et al., 2012	https://fiji.sc; RRID: SCR_002285
Cell^F	Olympus	http://www.dis-imaging.gr/OLYMPUS/software.html; RRID: SCR_014342
Other		
96-well MaxiSorp plates	Thermo Scientific	12565135
Glutathione agarose resin	Web Scientific	Cat#ABT 4B-GLU-100
Ni^{2+} agarose resin	Web Scientific	Cat#ABT 6BCL-QHNi-100
Source 15Q	GE Healthcare	Cat#17094701
Source 15S	GE Healthcare	Cat#17094401
SP Sepharose Fast Flow	GE Healthcare	Cat# 17072901
Superdex 75	GE Healthcare	Cat# 28989333
HisTrap HP	GE Healthcare	Cat# 17-5248-02
Protein A Sepharose CL 4B	GE Healthcare	Cat#17-0780-01
Protein G Sepharose 4 Fast Flow	GE Healthcare	Cat#17-0618-01

CONTACT FOR REAGENT AND RESOURCE SHARING

Further information and requests for reagents should be directed to Lead Contact Danny T. Huang (d.huang@beatson.gla.ac.uk).

EXPERIMENTAL MODEL AND SUBJECT DETAILS

Recombinant Proteins

All recombinant proteins were expressed in *Escherichia coli* BL21(λDE3) GOLD. Cells were grown at 37°C in Luria Bertani to an OD_{600} of 0.6–0.8 and induced with 0.2 mM isopropyl β-D-1-thiogalactopyranoside (IPTG) at 18–20°C overnight. To generate pTyr-Cbl variants, Cbl-encoding plasmids were co-expressed with plasmid encoding MBP-tagged *Mus* Src (84–526) as in Dou et al. (2012a). To generate heterodimeric RING E3s, plasmids encoding GST-tagged MDM2 or RNF2 were coexpressed with plasmids encoding His-tagged MDM4 or BMI1, respectively. ^{15}N-labeled UbV.E4B and E4B were obtained from M9 minimal media according to Cai et al. (1998). Briefly, cells were grown in 4 L of Luria Bertani media until OD_{600} reached 0.8-1.0, the cells were pelleted, washed

in 1xM9 media, all pelletes were combined into 1 L of M9 media and grown for 1 hr at 37°C to eliminate unlabelled precursors. Accordingly, the 1 L of M9 was supplmented with: 1 g of $^{15}NH_4Cl$, 4 g of glucose, 50 mg kanamycin, and essential neutrients then grown for an additional hour at 20°C. Finally, the cells were induced using a final concentration of 0.5 mM IPTG for 20 hr. ^{15}N-labeled Ub was prepared following the autoinducing system from Varadan et al. (2002). In brief, 10 mL of starter culture was added to 1 L of ^{15}N-autoinducing media containing: 1.42 g Na_2SO_4, 6.8 g KH_2PO_4, 7.1 g Na_2HPO_4, 1 mM $MgSO_4$, 1 g $^{15}NH_4Cl$, 100 mg ampicillin, 60 mg of Iron(III) citrate, 12.5 g glycerol, 2 g glucose, 5 g and lactose. The 1L culture was grown at 37°C for 20 hr and harvested.

Cell Culture and Transfection

HEK293T cells were cultured in DMEM and H1299 and HeLa cells were cultured in RPMI, all supplemented with 10% FBS, 20 mM L-glutamine, 100 units/ml penicillin, 0.1 mg/ml streptomycin and 6 mg/l gentamycin reagent solution (Invitrogen, USA). The cells were grown in monolayer at 37°C in 5% CO_2. The constructs were transfected into the respective cell lines using Lipofectamine-2000 (Thermo Fisher Scientific) following manufacturer's protocol. Cells were harvested 48 hr post transfection. The plasmids were transfected into cells seeded on 100 mm plates as follows: Myc-tagged UBE4B (5 μg) and HA-tagged UbV.E4B (5 μg) in Figure 2C; GFP-tagged p53 (1 μg) and HA-tagged UbV.pCBL (7.5 μg) or Ub74 (7.5 μg) in Figure 2D; Myc-tagged CBL (5 μg) and HA-tagged UbV.pCBL (5 μg) in Figure 3H; His-tagged Ub (1 μg), FLAG-tagged EGFR (2 μg) and HA-tagged UbV.pCBL (5 μg) or Ub74 (5 μg) in Figure 3I; HA-tagged UbV.pCBL (5 μg) or Ub74 (5 μg) in Figure 3J, Myc-tagged XIAP (5 μg) and HA-tagged UbV.XR (5 μg) in Figure 4G, UbV.XR (5 μg) or Ub74 (5 μg), His-tagged Ub (1 μg) in Figure 4H. In Figures 3J and 3L, 2.5 μg of each plasmid were transfected into cells seeded on 35 mm plates.

METHOD DETAILS

Selection of Ubiquitin Variants

The phage-displayed ubiquitin variant (UbV) library used in this study was re-amplified from Library 2 as previously described (Ernst et al., 2013). Protein immobilization and the following UbV selections were done according to established protocols (Tonikian et al., 2007). Specifically, purified RING E3s were coated on 96-well MaxiSorp plates by adding 100 μL of 1 μM proteins and incubating overnight at 4°C. Afterward, five rounds of selections using the phage-displayed UbV library were performed against immobilized proteins, as shown in Figure 1B including the following steps: (I) Within the phage pool, each phage particle displays a unique UbV and encapsulates the encoding DNA. (II) Protein-binding phage are captured with immobilized proteins. (III) Non-binding phage are washed away. (IV) Bound phage are amplified by infection of bacterial host (Zhang et al., 2016). After the fifth round of binding selections, individual phage with improved binding properties obtained from round 4 and round 5 were identified by phage ELISA (see below) and subjected to DNA sequencing of the phagemids to obtain UbV sequences.

ELISAs to Evaluate Binding and Specificity

Phage and protein ELISA against immobilized proteins under study was performed as previously described (Zhang et al., 2016). Briefly, GST-tagged RING/U-box domains from nine E3s (1 μM) were individually immobilized in microtiter plates (30 μL). Binding of phage was detected using anti-M13-HRP antibody and colorimetric development of TMB peroxidase substrate. For protein ELISA to measure the half maximal binding concentration (EC_{50}) of UbVs binding to purified RING E3s, two-fold serial dilutions of FLAG-tagged UbV or Ub (starting at 62.5 nM, 24 points, 30 μL) were added and incubated for 20 min at room temperature. Wells were washed and bound UbV was detected by anti-FLAG-HRP conjugate antibody and colorimetric development of TMB peroxidase substrate.

Generation of Constructs

New constructs were generated using standard PCR-ligation techniques and verified by automated sequencing. GST-tagged constructs were cloned into pGEX4T1 (GE Healthcare) modified with a TEV cleavage site (pGEX4T1 TEV), an N-terminal uncleavable His-tag (pGEX4T1 HG) or both as indicated (pGEX4T1 HG TEV); His-tagged constructs were cloned into pRSFDuet-1 (Merck Millipore) modified with a TEV cleavage site following the N-terminal His-tag (pRSFDuet TEV) and HisSmt3-tagged proteins were cloned into pET-28a (Merck Millipore) modified with a Ulp-1 cleavable hexahistidine Smt3-tag (pET-HisSmt3). Ub74 in pRSFDuet TEV and all the UbV constructs include DNA encoding the sequence GGS at the N terminus prior to Met1. Untagged Ub74 was cloned into pET-3a (Merck Millipore). The following mammalian constructs were generated for the study: pRK5 HA-Ub74, pRK5 HA-UbV.pCBL, pRK5 HA-UbV.XR, pcDNA3.1 Myc/His-CBL, pcDNA3.1 Myc/His-XIAP and pcDNA3.1 FLAG-EGFR.

Protein Purification

Following expression, bacterial cells were harvested by centrifugation and lysed with a microfluidizer. Cells expressing UbVs were resuspended in 25 mM Tris-HCl, pH 7.6, 500 mM NaCl, 5% glycerol, 15 mM imidazole, 5 mM β-mercaptoethanol (BME), 2.5 mM phenylmethylsulfonyl fluoride (PMSF); cells expressing pTyr-Cbl variants were resuspended in PBS mixed with 350 mM NaCl, 15 mM imidazole, 5 mM BME, 2.5 mM PMSF, 2 mM $NaVO_4$; cells expressing UbcH5B or UbcH5B S22R were resuspended in 100 mM NaCl, 50 mM MES, pH 6.5, 1 mM DTT; otherwise cells were resuspended in 25 mM Tris-HCl, pH 7.6, 200 mM NaCl, 15 mM imidazole, 5 mM BME, 2.5 mM PMSF.

To purify GST-tagged E4B and variants, RNF38 389–C, XR and variants, B2R, BIRC3 RING, BIRC7 RING, BRE1A, $pCBL_{47–435}$, pCBLR and variants, and CBLR used for UbV selection and/or SPR, clarified lysates were incubated with glutathione agarose resin for 1-2 hr at 4°C on a rotary shaker, washed in lysis buffer lacking PMSF and $NaVO_4$ and eluted in 50 mM Tris-HCl, pH 8.0, 200 mM NaCl, 10 mM glutathione, 5 mM DTT. GST-tagged pTyr-Cbl variants were further purified by anion exchange chromatography (Source 15Q) using a NaCl gradient in 50 mM Tris-HCl, pH 8.5. To purify GST-MDM2/His-MDM4 and GST-RNF2/His-BMI1, clarified lysates were incubated with Ni^{2+} agarose resin for 1-2 hr at 4°C on a rotary shaker, washed in lysis buffer lacking PMSF and $NaVO_4$ and eluted in 50 mM Tris-HCl, pH 8.0, 200 mM NaCl, 200 mM imidazole, 5 mM BME. The eluate was then incubated with glutathione agarose resin for 1-2 hr at 4°C on a rotary shaker, washed in lysis buffer lacking PMSF and $NaVO_4$ and eluted in 50 mM Tris-HCl, pH 8.0, 200 mM NaCl, 10 mM glutathione, 5 mM DTT.

To purify HisGST-tagged or HisSmt3-tagged UbVs, clarified lysates were applied to Ni^{2+}-affinity columns, washed in lysis buffer lacking PMSF and eluted in 25 mM Tris-HCl, pH 7.6, 500 mM NaCl, 200 mM imidazole, 5% glycerol, 5 mM BME. HisGST-tagged UbVs were further purified using glutathione affinity chromatography with elution in 50 mM Tris-HCl, pH 8.0, 500 mM NaCl, 5% glycerol, 10 mM glutathione, 5 mM DTT. Affinity tags were cleaved with TEV or Ulp-1 and removed by Ni^{2+}-affinity chromatography and protein was further purified using gel filtration chromatography (Superdex 75) in 25 mM Tris-HCl, pH 7.6, 500 mM NaCl, 5% glycerol, 1 mM DTT. For HisSmt3-tagged UbV.pCBL variants, anion exchange chromatography (Source 15Q) was performed instead of gel filtration chromatography.

To purify *Arabidopsis* Uba1 (Dou et al., 2012b), cells overexpressing the E1 were mixed with cells overexpressing GST-Ub. Clarified lysate from this mixture was incubated for 2 hr at 4°C with 5 mM $MgCl_2$ and 5 mM ATP, and purified using glutathione affinity chromatography with elution in 20 mM DTT. The eluate was further purified by anion exchange chromatography (Source 15Q).

For UbcH5B (Dou et al., 2012a) and UbcH5B S22R (Dou et al., 2012b), clarified lysate was diluted to 50 mM NaCl, 50 mM MES, pH 6.5, 1 mM DTT, loaded onto an SP Sepharose Fast Flow column, washed with 50 mM MES, pH 6.5, 50 mM NaCl, 1 mM DTT, and eluted with 50 mM MES, pH 6.5, 200 mM NaCl, 1 mM DTT. The eluate was subsequently diluted to 50 mM MES, pH 6.5, 50 mM NaCl, 1 mM DTT, loaded onto a Source 15S column, eluted with a NaCl gradient in 50 mM MES, pH 6.5 and further purified by gel filtration chromatography (Superdex 75) in 25 mM Tris-HCl, pH 7.6, 150 mM NaCl, 1 mM DTT.

To prepare ^{32}P-Ub (Huang et al., 2008), clarified lysate from cells expressing GST-tagged 2TK-Ub was applied to a glutathione affinity column, cleaved with TEV, passed back over a glutathione affinity column to remove the tag, and applied to a Superdex 75 gel filtration column in 25 mM Tris-HCl pH 7.6, 150 mM NaCl, 1 mM DTT. To generate ^{32}P-Ub, purified 2TK-tagged Ub was incubated with γ-^{32}-P-ATP in 15 mM Tris-HCl pH 7.6, 100 mM NaCl, 12 mM $MgCl_2$, 1 mM DTT for 2 hr at 23°C.

To purify E4B (HisGST-tagged) or $E4B_{1097–C}$ (HisSmt3-tagged), clarified lysates were applied to Ni^{2+}-affinity columns, washed in lysis buffer lacking PMSF and $NaVO_4$ and eluted in 25 mM Tris-HCl, pH 7.6, 500 mM NaCl, 200 mM imidazole, 5 mM BME. Affinity tags were cleaved with TEV or Ulp-1 and removed by Ni^{2+}-affinity pass-back and protein was further purified using gel filtration chromatography (Superdex 75) in 25 mM Tris-HCl pH 7.6, 150 mM NaCl, 1 mM DTT.

To purify $pCBL_{47–435}$ (Dou et al., 2012a), clarified lysate was applied to a Ni^{2+}-affinity column, washed in PBS mixed with 350 mM NaCl, 10 mM imidazole, 5 mM BME, and eluted in PBS mixed with 350 mM NaCl, 250 mM imidazole, 5 mM BME. Eluate was then applied to a glutathione Sepharose column, eluted in 50 mM Tris-HCl, pH 8.5, 200 mM NaCl, 10 mM glutathione, 5 mM DTT, cleaved with thrombin and applied to a Ni^{2+}-affinity column to remove the cleaved tag. Subsequently, protein was purified by anion exchange chromatography (Source 15Q) with a NaCl gradient in 50 mM Tris-HCl, pH 8.5 and gel filtration chromatography (Superdex 75) in 25 mM Tris-HCl, pH 7.6, 150 mM NaCl, 1 mM DTT.

pTyr-Cbl variants were purified using the above protocol, but after elution with glutathione, protein was cleaved with thrombin and then purified by anion exchange chromatography (Source 15Q) followed by gel filtration chromatography (Superdex 75). To purify CBLR, the above protocol was used but instead of eluting with glutathione buffer, protein was cleaved with thrombin directly from the beads.

To purify His-tagged Ub74, Ub Q31R, and UbV.XR variants, clarified lysates were incubated with Ni^{2+} agarose resin for 1-2 hr at 4°C on a rotary shaker. Bound protein was washed in lysis buffer lacking PMSF and cleaved with TEV.

Complex of $pCBL_{47–435}$ and UbV.pCBL was obtained by expressing GST-tagged $pCBL_{47–435}$ as described above and then mixing the resuspended cells with cells overexpressing His-tagged UbV.pCBL. Complex was purified by Ni^{2+}-affinity and then glutathione affinity chromatography followed by cleavage with thrombin to remove the GST-tag. Protein was then loaded onto HisTrap HP columns, cleaved with TEV and further purified by gel filtration chromatography.

For XR and its variants, and XIAP, clarified lysate was loaded onto Ni^{2+}-affinity columns, washed in lysis buffer lacking PMSF, and eluted in 25 mM Tris-HCl, pH 7.6, 200 mM NaCl, 200 mM imidazole, 5 mM BME. Eluted protein was loaded onto glutathione-resin, washed in 25 mM Tris-HCl, pH 7.6, 150 mM NaCl, 5 mM BME, and eluted in 25 mM Tris-HCl, pH 7.6, 150 mM NaCl, 10 mM glutathione and 5 mM DTT. The eluted protein was cleaved with TEV protease while dialyzing against 25 mM Tris-HCl, pH 7.6, 150 mM NaCl, 5 mM BME, passed over a Ni^{2+}-affinity column to remove the tag and applied onto a size-exclusion chromatography column (Superdex 75) equilibrated in 25 mM Tris-HCl, pH 7.6, 150 mM NaCl, 1 mM DTT.

mSMAC containing a C-terminal Protein Kinase A recognition sequence (RRAVS) was purified by loading clarified lysates onto Ni^{2+} affinity columns, washing in lysis buffer lacking PMSF, and elution in 25 mM Tris-HCl, pH 7.6, 200 mM NaCl, 200 mM Imidazole, 5 mM BME. The eluted protein was then purified by gel filtration chromatography (Superdex 75) in 25 mM Tris-HCl, pH 7.6, 150 mM NaCl.

Preparation of untagged Ub74 and Ub variants was based on Volk et al. (2005). Cells expressing untagged Ub variants were lysed and then 70% perchloric acid was added dropwise to the clarified lysate in an ice bath until a final concentration of 0.4%. This mixture was centrifuged and the supernatant was dialyzed in a 3.5 kDa MWCO membrane against 50 mM ammonium acetate, pH 4.5 at 4°C for 16 hr. The contents were loaded onto a 16/10 SP FF column (GE Life Sciences) and eluted using a 0%–40% gradient over 15 column volumes of 50 mM ammonium acetate, 1 M NaCl, pH 4.5. Ub fractions were pooled, concentrated and loaded onto a 26/60 Superdex 75 gel filtration column for exchange into PBS buffer.

K48-diUb was prepared enzymatically by following Varadan et al. (2002). The 2 mL reaction containing 20 mg of Ub K48R, 20 mg of Ub74, 10 μM of GST-UBE2K, 1 μM E1, 15 mM ATP, 5 mM TCEP, 10 mM $MgCl_2$ in 50 mM Tris-HCl, pH 8.0 buffer was allowed to react at 30°C for 20 hr. K48-diUb was purifed by applying the 2 mL reaction to a 26/60 Superdex 75 gel filtration column in PBS buffer at pH 7.4.

Stably conjugated UbcH5B C85K S22R–Ub was generated as described previously (Dou et al., 2013): in brief, purified *Arabidopsis* Uba1, UbcH5B C85K S22R and His-GST-Ub were mixed in 50 mM Tris-HCl, pH 9.0, 200 mM NaCl, 10 mM $MgCl_2$ and 10 mM ATP for 1 day at 30°C; the mixture was applied to a Ni^{2+}-agarose column, cleaved with TEV, and further purified by cation exchange (Source 15S) followed by gel filtration chromatography (Superdex 75).

Absorbance at 280 nm was measured to determine the concentrations of Ub variants (including the UbVs) and UbV.pCBL-$pCBL_{47–435}$ complex based on molar extinction coefficients calculated from the relevant sequences using Expasy's ProtParam (Gasteiger et al., 2005). Two concentrations were calculated for UbV.XR_D based on treating it as a dimer or a monomer. Other protein concentrations were determined by Bradford assay using BSA as a standard.

SPR Binding Assays

All SPR experiments were performed at 25°C on a Biacore T200 with a CM-5 chip (GE Healthcare). Anti-GST was coupled onto a CM-5 chip and GST-tagged E3s were captured to a level of 1,000–2,000 response units (Dou et al., 2012a). UbVs and UbcH5B S22R C85K–Ub were serially diluted in running buffer containing 25 mM Tris-HCl, pH 7.6, 150 mM NaCl, 0.1 mg/ml BSA, 1 mM DTT and 0.005% (v/v) Tween-20. For experiments performed in the presence of UbVs and UbcH5B S22R C85K–Ub, UbcH5B S22R C85K–Ub was serially diluted in running buffer containing 10 μM of the indicated UbV.XR variant in Table 1 and Figure S1. Binding was measured at the concentration ranges indicated in Figure S1. Data reported are the difference in signal between GST-E3 variants and GST alone.

Solution NMR Experiments

All NMR experiments were carried out in 25mM sodium phosphate, 100mM NaCl, 5% (v/v) D_2O buffer on a Bruker Avance III 600MHz spectrometer equipped with a cryogenic probe at 298K. ^{15}N-HSQC spectra were acquired with 8 scans over 128 points in the F2 dimension, with a spectra width of 18ppm. NMR data was processed using Top Spin v3.1 and analyzed in UCSF Sparky. Assignment for human E4B was taken from BMRB-16623 and confirmed with 2D TOCSY. Chemical shift perturbations (CSPs) were calculated from (Equation 1):

$$CSP = \left[(\delta_{HA}\text{-}\delta_{HB})^2 + ((\delta_{NA} - \delta_{NB})/5)^2\right]^{1/2} \quad \text{Equation 1}$$

Where the δ_H and δ_N are the proton and nitrogen chemical shifts, respectively for a given residue.

Analytical Gel Filtration

UbV.XR and variants were applied onto a Superdex 75 10/300 column (GE Healthcare) pre-equilibrated in 25 mM TrisHCl, pH 7.6, 150 mM NaCl, 1 mM DTT at constant flow-rate.

Crystallization

UbV.pCBL- $pCBL_{47–435}$ Complex

A 3-fold molar excess of ZAP70 peptide (Dou et al., 2012a) was added to UbV.pCBL- $pCBL_{47–435}$ complex (10 mg/ml) for crystallization. Crystals were obtained by sitting drop vapor diffusion from the Morpheus screen condition 62 (0.1 M monosaccharides, 0.1 M Buffer 1 pH 6.5, 50% Precipitant 2) using a 1:1 ratio of protein:reservoir. No further cryoprotection was required.

$E4B_{1097–C}$

$E4B_{1097–C}$ (1000 μM) and UbV.E4B (1000 μM) were mixed in a 1:1 molar ratio and dialyzed against 150 mM NaCl, 25 mM Tris-HCl, pH 7.6. Crystals of $E4B_{1097–C}$ alone were obtained from this mixture by hanging drop vapor diffusion using a 1:1 ratio of protein:reservoir in Bis-Tris pH 5.5, 1.9 M ammonium sulfate and cryoprotected in mother liquor with an additional 20% glycerol.

XR-UbV.XR Complex

XR (1000 μM) and UbV.XR (1000 μM) were mixed in a 1:1.2 molar ratio. Crystals were obtained by sitting drop vapor diffusion in the Classics Suite condition 44 (0.1 M NaHEPES pH 7.5, 1.4 M tri-Na citrate) using a 2:1 ratio of protein:reservoir and cryoprotected in mother liquor with an additional 20% ethylene glycol. Crystals of UbV.XR_D (600 μM) were obtained by sitting drop vapor diffusion in JCSG+ condition 30 (0.2 M Zinc acetate, 0.1 M phosphate-citrate pH 4.2, 40% PEG 300) and swiftly pulled through 100% paraffin oil before snap-freezing in liquid N_2.

Data Collection and Structure Determination

Data were collected at Diamond Light Source beamlines I03 and I04, and processed using the xia2 pipeline (Winter, 2010), including XDS (Kabsch, 2010), POINTLESS (Evans, 2006), AIMLESS (Evans and Murshudov, 2013), and autoPROC (Vonrhein et al., 2011). Models of XR generated by modifying BIRC3 RING (3EB5; Mace et al., 2008), $pCBL_{47-435}$ (4A4C; Dou et al., 2012a), or UBE4B U-box (3L1Z; Benirschke et al., 2010) and Ub (from 4V3K; Buetow et al., 2015), were used as initial search models for PHASER (McCoy et al., 2007). The structures were refined in BUSTER (Bricogne et al., 2016) or PHENIX (Adams et al., 2010), and manually inspected in COOT (Emsley et al., 2010). TLS parameterisation was used throughout. The final models were validated using MOLPROBITY (Chen et al., 2010). All data processing and refinement statistics are presented in Table 2. Superimpositions and protein surface areas were respectively calculated in LSQMAN (Kleywegt, 1996) and PISA (Krissinel and Henrick, 2007), and figures were made in PyMOL.

Autoubiquitination Assays

Autoubiquitination assays were performed at 23°C in 50 mM Tris-HCl, pH 7.6, 50 mM NaCl, 5 mM $MgCl_2$, 5 mM ATP, 1 mM DTT, 0.3 U/ml inorganic pyrophosphatase, 0.3 U/ml creatine kinase and 5 mM creatine phosphate, *Arabidopsis* Uba1 (0.5 μM), UbcH5B (5 μM), and ^{32}P-Ub (100 μM) with the following: GST-pCBLR (0.75 μM) and UbV.pCBL (15 μM) or Ub74 (15 μM), GST-E4B (5 μM) and UbV.E4B (100 μM) or Ub74 (100 μM), or GST-XR (2.5 μM) and UbV.XR (50 μM) or Ub74 (50 μM) where the final reaction concentrations are given in parenthesis. Reactions were quenched at indicated time points with 2X loading dye containing 500 mM DTT and resolved by SDS-PAGE. Gels were dried and visualized by autoradiography.

Single-Turnover Lysine Discharge Assays

UbcH5B S22R (10 μM) was charged with *Arabidopsis* Uba1 (0.4 μM) and ^{32}P-Ub (11.2 μM) for 15 min at 23°C in 50 mM Tris-HCl (pH 7.6), 50 mM NaCl, 5 mM MgCl2, 5 mM ATP, 1mM DTT, BSA (1 mg/ml). For Figures 4B–4D, $UbV.XR_D$ and $UbV.XR_M$ were included in the charge. Charging was stopped by incubating the reaction with 0.01 U/ml apyrase and 30 mM EDTA for 1-2 min. Discharge was initiated by the addition of a mixture containing 50 mM Tris-HCl (pH 7.6), 50 mM NaCl, BSA (1 mg/ml), L-lysine (150 mM), E3 and Ub74 or UbV. Concentrations of E3 and Ub74 or UbV were as follows: for Figure 2A, E4B (1 μM), UbV.E4B and Ub74 as indicated; for Figures 3A and 3G, pCBLR (60 nM), CBLR (1 μM), pCBL-B (75 nM), pCBL-C (42 nM), UbV.pCBL (10 μM) and Ub74 (10 μM); for Figure 3F, pCBLR (60 nM), UbV.pCBL variants (2 μM) and Ub74 (2 μM); for Figure 4B, XR (350 nM), $UbV.XR_D$ (5 μM using dimer molar extinction coefficient or 10 μM using monomer molar extinction coefficient), $UbV.XR_M$ (10 μM) and Ub74 (10 μM); for Figure 4C, XR (200 nM) and $UbV.XR_D$ and $UbV.XR_M$ as indicated; for Figure 4D, XR (200 nM) and $UbV.XR_D$ and $UbV.XR_M$ were at 1.11 μM based on the molar extinction coefficient of a monomer and discharge at 1.5 min quantified; for Figure 4E, XR variants (350 nM), B2R (75 nM), $UbV.XR_D$ (5 μM), and Ub74 (10 μM); for Figure 5C, XR (350 nM), UbV.XR variants (10 μM for monomers or 5 μM for dimers), and Ub74 (10 μM); for Figure 5D, XR (350 nM), UbV.XR variants (10 μM for monomers or 5 μM for dimers), and Ub74 (10 μM); for Figure 5H, XR (350 nM), $UbV.XR_D$ (5 μM), and Ub74 (10 μM). Reactions were quenched at indicated times with 4X loading dye and resolved by SDS-PAGE. Gels were dried and visualized by autoradiography. For Figure 5H, Ub or Ub Q31R (11.2 μM) were used instead of ^{32}P-Ub and reactions were visualized by staining with InstantBlue (Expedeon). Final concentrations are in parenthesis except for UbcH5B S22R and ^{32}P-Ub, Ub or Ub Q31R, which were 5 μM and 5.6 μM, respectively. For assays in which effects or activities of several mutants were compared (e.g., UbV.pCBL, UbV.pCBL Y66T Y68H and UbV.pCBL Q8L P11K or XR, XR T489E and XR F490R), concentrations were normalized to unmutated variant or wild-type protein based on measured intensities of Coomassie stained protein bands following separation by SDS-PAGE.

Single-Turnover Ub Transfer Reactions

UbcH5B S22R (5 μM) was charged with ^{32}P-Ub (5.6 μM) and stopped as described for lysine discharge reactions but without BSA. Ub transfer was initiated by the addition of: $pCBL_{47-435}$ (1 μM) and UbV.pCBL (10 μM) or Ub74 (10 μM); GST-E4B (5 μM) and UbV.E4B or Ub74 at indicated concentrations; XIAP (2 μM) and $UbV.XR_D$ (5 μM using dimer molar extinction coefficient or 10 μM using monomer molar extinction coefficient) or Ub74 (10 μM); XIAP (2 μM), mSMAC (20 μM) and $UbV.XR_D$ (5 μM using dimer molar extinction coefficient or 10 μM using monomer molar extinction coefficient) or Ub74 (10 μM). Reactions were quenched at indicated times with 4X loading dye, resolved by SDS-PAGE, dried and visualized by autoradiography. Final reaction concentrations are in parenthesis.

Chemicals and Antibodies for Cell Culture

The chemicals used include hEGF, etoposide and MG132. Etoposide and MG132 were dissolved in DMSO and used at final concentrations of 75 μM and 50 μM, respectively. Where indicated, transfected cells were treated with etoposide for 18 hr and with MG132 for 4 hr prior to harvesting. Human EGF (hEGF) was dissolved in sterile PBS and used at a final concentration of 100 ng/ml for 10 min unless otherwise stated in the figures. Before EGF treatment, the cells were serum starved for 24 hr. The following primary antibodies were used: rabbit anti-FLAG, mouse anti-HA, mouse anti-Myc tag, mouse anti-GFP, mouse anti-Smac, mouse anti-EEA1, rabbit anti-EGFR, rabbit anti-phospho-Akt, mouse anti-Akt, rabbit anti-phospho-Erk1/2, mouse Erk1/2, rabbit anti-ubiquitin, goat anti-Actin and mounting medium with DAPI antibody. The secondary antibodies were donkey anti-goat IRDye 800CW, goat anti-rabbit IRDye 800CW, and goat anti-mouse IRDye 800CW, goat anti-rabbit Alexa Fluor 488, goat anti-mouse Alexa Fluor 594.

Co-immunoprecipitation and Immunoblotting

The whole cell lysates (WCL) for western blotting were prepared in 50 mM Tris-HCl, pH 7.5, 150 mM NaCl, 1 mM EDTA, 1% IGEPAL CA-630, 10% glycerol 1.0 mM DTT and cOmplete protease inhibitor cocktail as described previously (Ahmed et al., 2015). Lysates for immunoprecipitation (IP) were prepared in 50 mM NaHEPES, pH 7.2, 150 mM NaCl, 10% glycerol, 1% IGEPAL CA-630, 1 mM EDTA, 0.5 mM DTT, 10 mM PMSF and cOmplete protease inhibitor cocktail using the same procedure. For immunoprecipitation, 1 mg of freshly prepared whole cell lysates were incubated with 25 μL (50% slurry) 3:1 mixture of Protein A Sepharose CL 4B and Protein G Sepharose 4 Fast Flow beads for pre-clearing at 4°C for 30 min on a rotatory shaker. The supernatants were then collected and incubated with the indicated antibodies at 4°C overnight on a rotatory shaker. The next day, 35 μL (50% slurry) beads were added to the samples and incubated for 2 hr at 4°C. The beads were microfuged at 2400 rpm for 2 min and washed once with IP lysis buffer and twice with IP wash buffer (same as IP lysis buffer except for 200 mM NaCl and 1.0 mM DTT). Beads were incubated with 40 μL 2X loading dye at 95°C for 10 min to elute the immunoprecipitated proteins. For immunoblotting from whole cell lysates, 50 μg of protein were loaded per lane. Proteins were separated using SDS-PAGE under reducing conditions and transferred onto nitrocellulose membrane (GE Healthcare Life Sciences). Blots were blocked with 5% BSA, washed with TBST and incubated with the respective primary antibodies indicated in Figures 2, 3, and 4 at 4°C overnight. The following day, the blots were visualized using an Odyssey CLx Imaging System (LI-COR Biosciences) after incubation with the secondary antibodies.

Ubiquitination Assays in Cells

For cell-based ubiquitination assays, HEK293T cells were transfected with CMV-driven plasmids expressing His-Ub, FLAG-EGFR, HA-UbV.pCBL, HA-UbV.XR and HA-Ub74 as indicated in Figures 3 and 4. Cells were harvested 48 hours post- transfection following the addition of EGF, etoposide and MG132 as indicated. The WCLs were prepared with IP lysis buffer and 1 mg protein was incubated with Ni-NTA resin for 4 hr at 4°C. The beads were then washed once in IP lysis buffer, three times in Wash buffer I (8 M Urea, 1% SDS in PBS) and once in wash buffer II (1% SDS in PBS). Pulled-down proteins were eluted in 40 μL 2X SDS sample loading buffer following incubation at 95°C for 10 min, separated using SDS-PAGE and probed for ubiquitinated adducts with immunoblotting.

Immunocytochemistry

The protocol described in Ahmed et al., 2015 was followed. Briefly, the cells were fixed with 4% paraformaldehyde, permeabilized with 0.5% Triton X-100, and blocked with 3% BSA in PBS. This was followed by overnight incubation with primary antibodies at 4°C, then secondary antibodies for 1 h at room temperature under dark conditions. The images were captured with a DP71 camera mounted on a BX51 fluorescence microscope (Olympus) using Cell^F software at 100X (oil) magnification. Post acquisition, the images were processed to generate the merged (red, green and blue channels) figures with Fiji software.

cDNA Synthesis and Quantitative Real-Time PCR

TRIzol method was used to extract RNA from samples. RNA samples were checked for quality using A260/280 measurements and agarose gel electrophoresis, cDNA were synthesized from the high quality RNA using the High capacity cDNA reverse transcription kit (ThermoFisher Scientific). Quantitative PCR reaction mixtures contained of 400 ng cDNA and subsequently the reaction was carried out in an Applied Biosystems 7500 Fast Real Time PCR system (ThermoFisher Scientific) using SYBR Green master mix (ThermoFisher Scientific). In all the experiments, 18S rRNA was used as an internal control. Data collected from the instrument were plotted in PRISM.

QUANTIFICIATION AND STATISTICAL ANALYSIS

For ELISAs and curves generated in Figure 1, EC_{50} values were calculated using the GraphPad Prism software with the built-in equation formula (non-linear regression curve). For SPR assays, the data were analyzed by steady-state affinity analysis using Biacore T200 BIAevaluation software (GE Healthcare) and Scrubber2 (BioLogic Software); data are presented as mean ± SEM in Table 1 and the number of replicates (n) for each K_D measurement is two (Figure S1). For Figure 4D, gels were exposed to a storage phosphor screen (Fujifilm BAS-IP SR 2025) and the screen was read with a Typhoon FLA 7000 laser scanner (GE Healthcare). ImageQuantTL 8.1 was used to quantify the intensity of each band (I) and the % UbcH5B~Ub remaining was calculated using the following equation:

$$\%\ \text{UbcH5B} \sim \text{Ub remaining} = 100 * I_{t=1.5\ \text{min}} / I_{t=0\ \text{min}}$$

Data are presented as an average ± SD based on four replicates for each measurement.

DATA AND SOFTWARE AVAILABILITY

The accession numbers for the atomic coordinates of the structures determined in this work are as follows: PDB: 5O6T (XR-UbV.XR complex), PDB: 5O6S (UbV.XR), PDB: 5O76 (ZAP70 peptide- $CBL_{47–435}$-UbV.pCBL complex) and PDB: 5O75 ($E4B_{1097–C}$). Raw gel images and ^{32}P-film scans have been deposited to Mendeley Data (https://doi.org/10.17632/hxd3cyxzrc.1).

Molecular Cell, Volume *68*

Supplemental Information

A General Strategy for Discovery of Inhibitors and Activators of RING and U-box E3 Ligases with Ubiquitin Variants

Mads Gabrielsen, Lori Buetow, Mark A. Nakasone, Syed Feroj Ahmed, Gary J. Sibbet, Brian O. Smith, Wei Zhang, Sachdev S. Sidhu, and Danny T. Huang

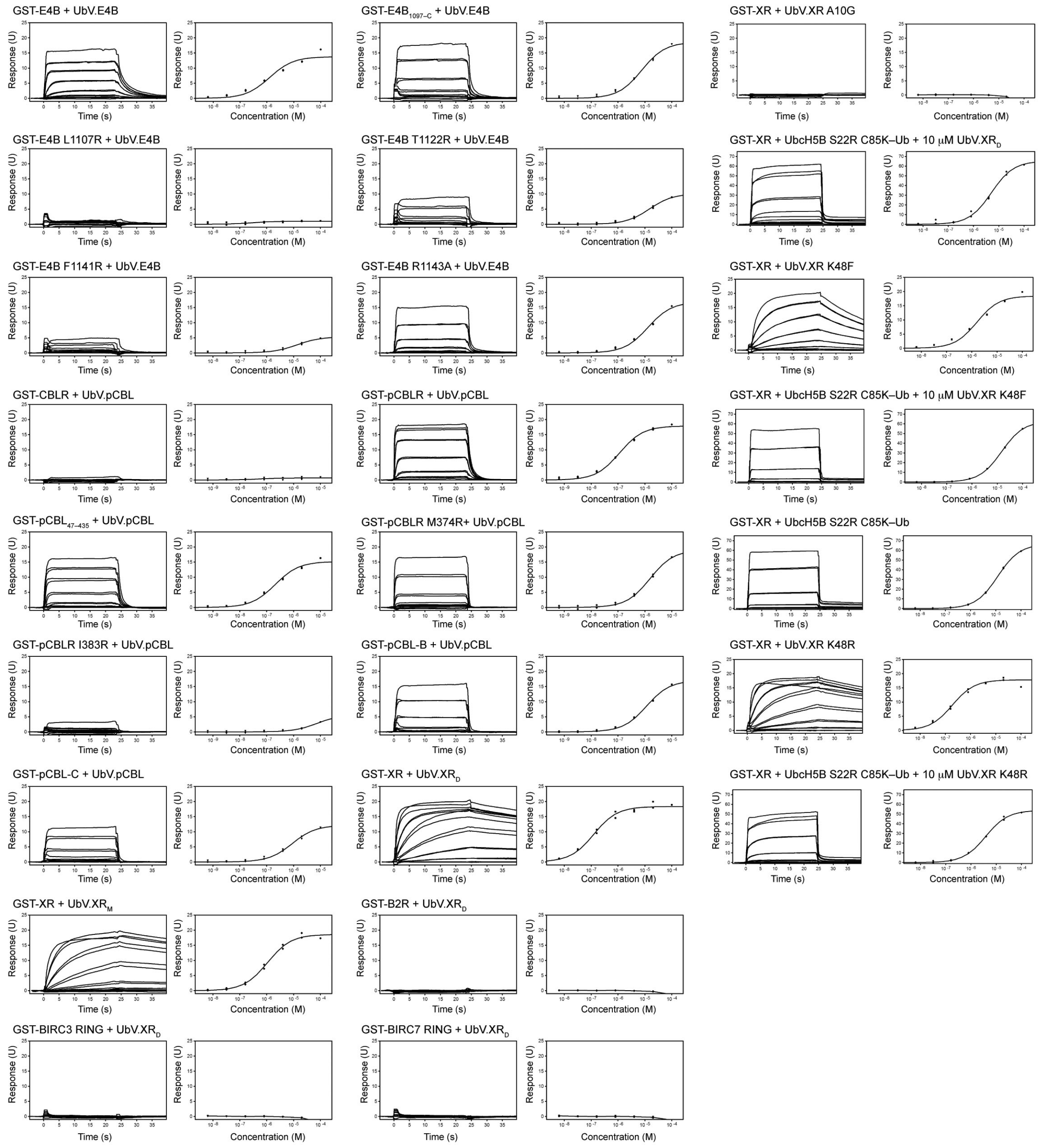

Figure S1 SPR analyses of GST-E3s and analyte binding affinities, related to Table 1. Representative sensorgrams (left) and binding curves (right) for GST-RING or U-box domains and their respective analytes as indicated above each set of sensorgram and binding curve. For GST-XR + UbcH5B S22R C85K–Ub + UbV.XR$_D$, UbV.XR K48R and UbV.XR K48F analyses, the concentration of UbcH5B S22R C85K–Ub was varied while the concentration of each UbV.XR variant was maintained at 10 μM. n=2 for each binding curve.

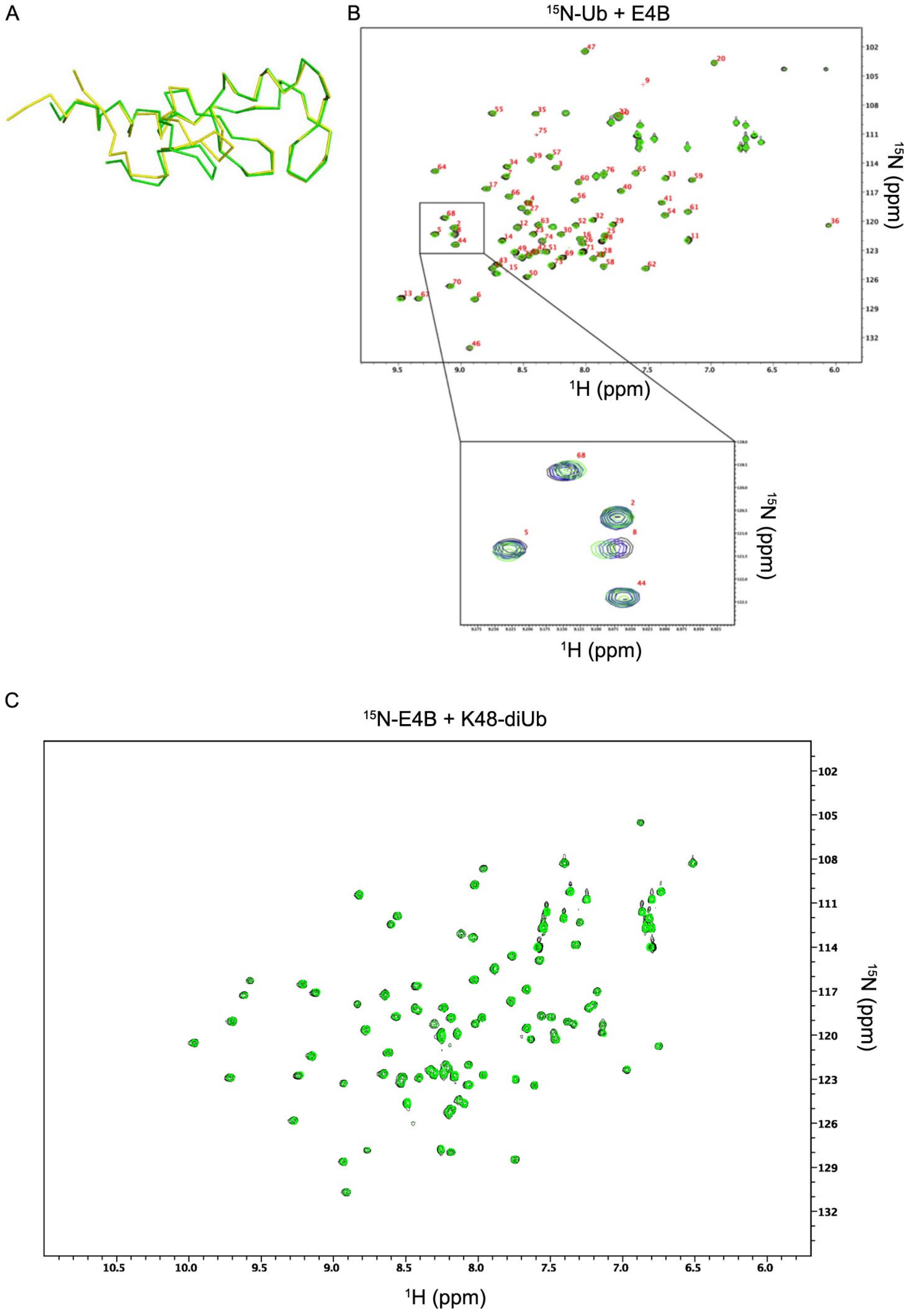
A
B
15N-Ub + E4B
1H (ppm)
15N (ppm)
C
15N-E4B + K48-diUb
1H (ppm)
15N (ppm)

Figure S2 Analyses of E4B structure and binding to Ub to Figure 2. (**A**) Overlay of $E4B_{1097-C}$ (green) with UBE4B (yellow) from PDBID 3L1Z (r.m.s.d. 0.631 Å across 431 atoms). (**B**) Chemical shift perturbation data for Ub-E4B interactions. ^{1}H-^{15}N HSQC spectra for ^{15}N-Ub alone (black) and with E4B (green, [^{15}N-Ub]:[E4B]=4.98). Inset shows close-up of selected peaks of ^{15}N-Ub alone (black) and with E4B (blue, [^{15}N-Ub]:[E4B]=1.29; green, [^{15}N-Ub]:[E4B]=4.98). (**C**) Chemical shift perturbation data for K48-diUb interactions with E4B. ^{1}H-^{15}N HSQC spectra for ^{15}N-E4B alone (black) and with K48-diUb (green, [K48-diUb]:[^{15}N-E4B]=1.87).

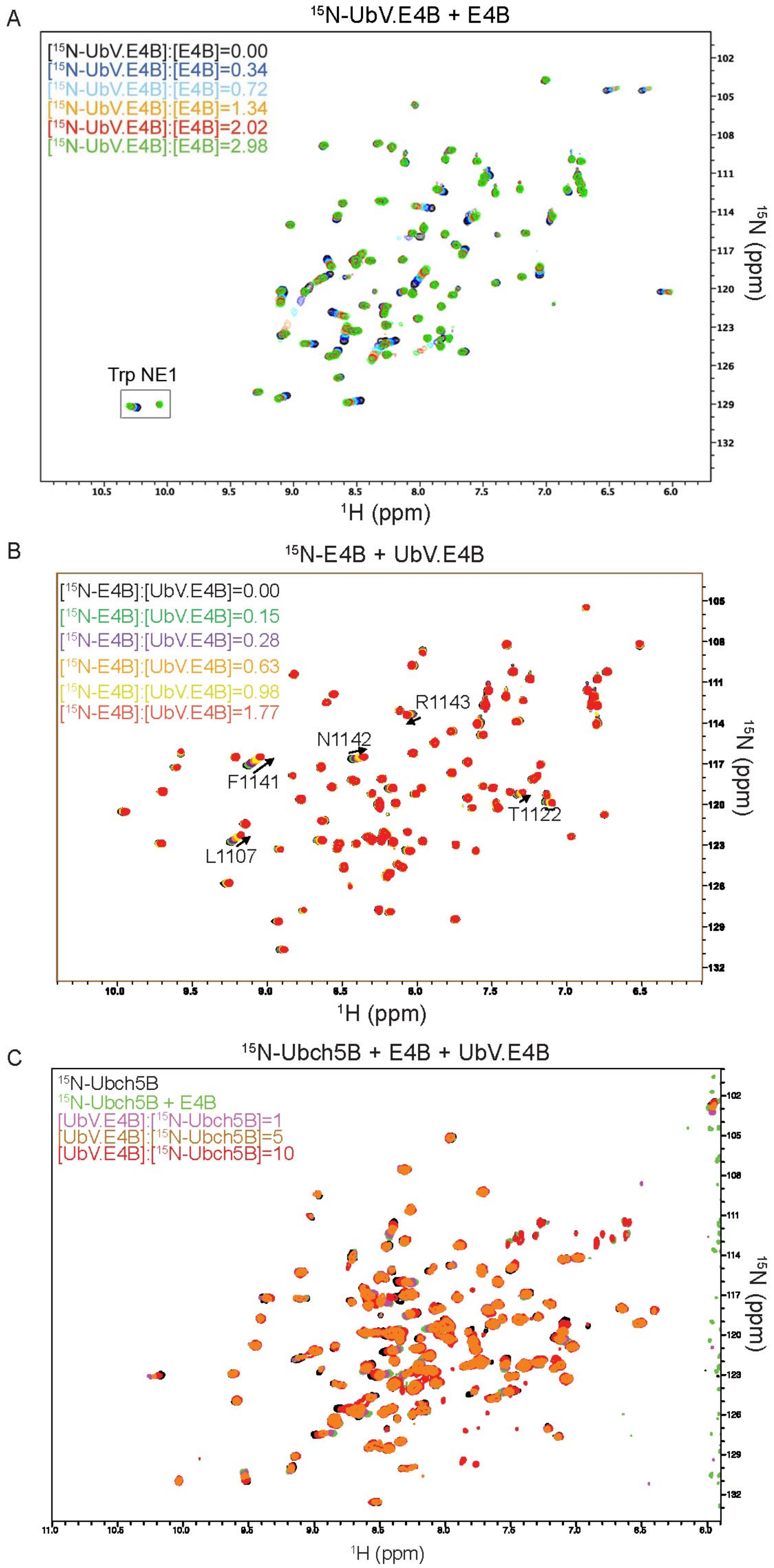
A
^{15}N-UbV.E4B + E4B
[^{15}N-UbV.E4B]:[E4B]=0.00
[^{15}N-UbV.E4B]:[E4B]=0.34
[^{15}N-UbV.E4B]:[E4B]=0.72
[^{15}N-UbV.E4B]:[E4B]=1.34
[^{15}N-UbV.E4B]:[E4B]=2.02
[^{15}N-UbV.E4B]:[E4B]=2.98
Trp NE1
^{1}H (ppm)
^{15}N (ppm)
B
^{15}N-E4B + UbV.E4B
[^{15}N-E4B]:[UbV.E4B]=0.00
[^{15}N-E4B]:[UbV.E4B]=0.15
[^{15}N-E4B]:[UbV.E4B]=0.28
[^{15}N-E4B]:[UbV.E4B]=0.63
[^{15}N-E4B]:[UbV.E4B]=0.98
[^{15}N-E4B]:[UbV.E4B]=1.77
R1143
N1142
F1141
T1122
L1107
^{1}H (ppm)
^{15}N (ppm)
C
^{15}N-Ubch5B + E4B + UbV.E4B
^{15}N-Ubch5B
^{15}N-Ubch5B + E4B
[UbV.E4B]:[^{15}N-Ubch5B]=1
[UbV.E4B]:[^{15}N-Ubch5B]=5
[UbV.E4B]:[^{15}N-Ubch5B]=10
^{1}H (ppm)
^{15}N (ppm)

Figure S3 Analyses of E4B binding to UbV.E4B, related to Figure 2. (**A**) Chemical shift perturbation data for UbV.E4B in UbV.E4B-E4B interactions. ^{1}H-^{15}N HSQC spectra for ^{15}N-UbV.E4B alone (black) and with selected molar ratios of E4B indicated by different colors. A 281 μM sample of ^{15}N-UbV.E4B was titrated with a 3.65 mM solution of E4B until a molar ratio of 3:1 was reached. At the endpoint, the concentrations of ^{15}N-UbV.E4B and E4B were 228 μM and 682 μM, respectively. (**B**) Chemical shift perturbation data for E4B in UbV.E4B-E4B interactions. ^{1}H-^{15}N HSQC spectra for ^{15}N-E4B alone (black) and with selected molar ratios of UbV.E4B indicated by different colors. A 150 μM sample of ^{15}N-E4B was titrated with a 355 μM solution of UbV.E4B until a molar ratio of 1.77:1 was achieved. At the endpoint, the concentrations of ^{15}N-E4B and UbV.E4B were 86 μM and 152 μM, respectively. (**C**) Chemical shift perturbation data for UbcH5B in competition assays with UbV.E4B for binding to E4B. ^{1}H-^{15}N HSQC spectra for ^{15}N-UbcH5B alone (black, 150 μM), following addition of a one molar equivalent of a 3.5 mM solution of E4B (green) and subsequently titrated with selected molar ratios of a 0.355 mM solution of UbV.E4B indicated by different colors until [UbV.E4B]:[^{15}N-UbcH5B] was 10:1. At the endpoint, the concentrations of ^{15}N-UbcH5B, E4B and UbV.E4B were 30, 30 and 300 μM, respectively.

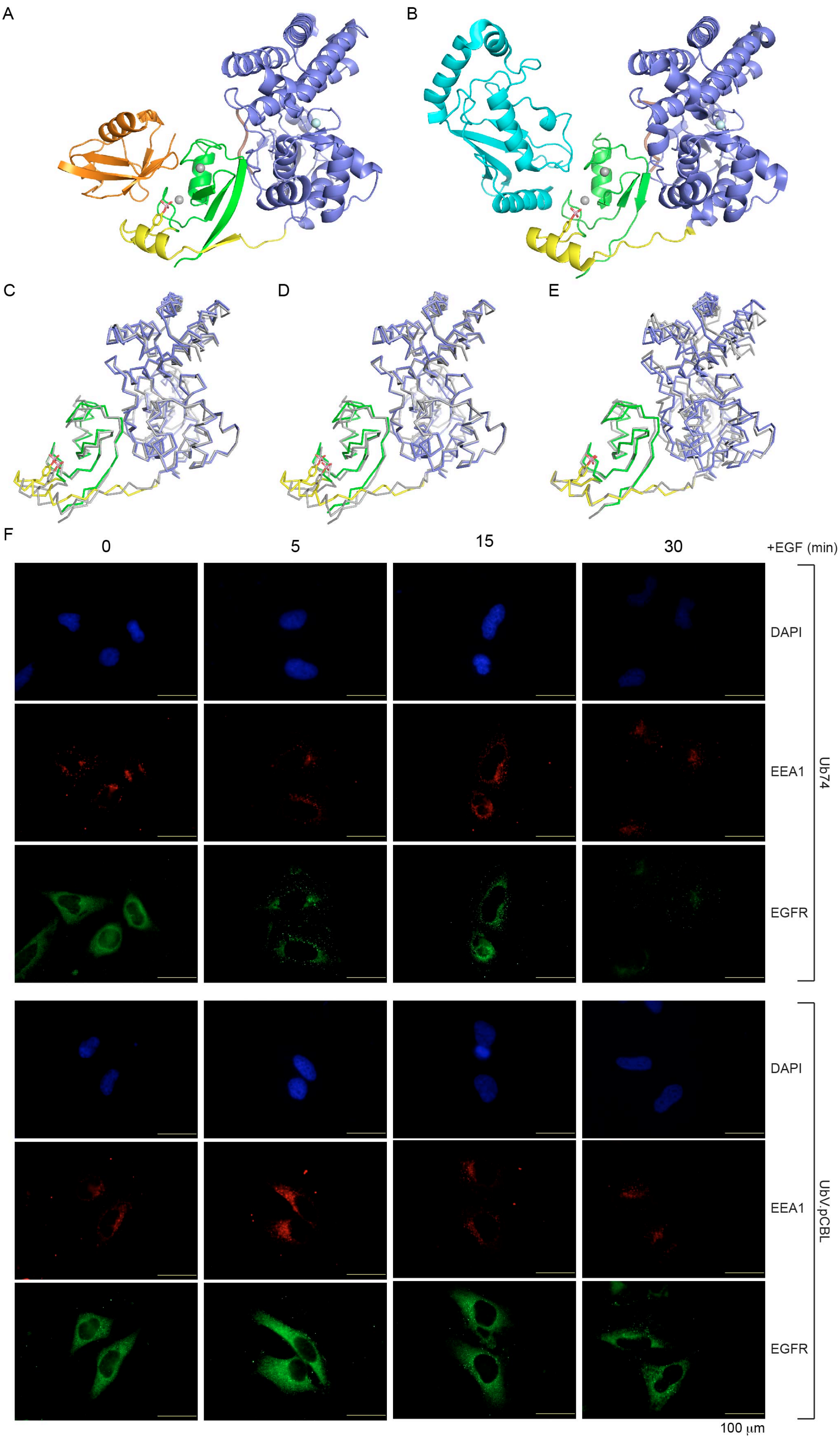
A
B
C
D
E
F
0
5
15
30
+EGF (min)
DAPI
EEA1
EGFR
Ub74
DAPI
EEA1
EGFR
UbV.pCBL
100 µm

Figure S4 UbV.pCBL effects on pCBL, related to Figure 3. (**A**) Cartoon representation of ZAP70 peptide-pCBL$_{47–435}$-UbV.pCBL colored as in **Figure 3** with pTyr371 shown in sticks. (**B**) Cartoon representation of ZAP70 peptide-pCBL$_{47–435}$-UbcH5B (PDBID 4A4B) with ZAP70 peptide and pCBL$_{47–435}$ colored as in **Figure 3** and UbcH5B colored cyan. pTyr371 is shown in sticks. **(C)** Ribbon overlay of pCBL$_{47–435}$ from the complexes shown in **A** and **B** (r.m.s.d. 1.37 Å across 379 Cα atoms). **(D)** Ribbon overlay based on the TKBD of pCBL$_{47–435}$ from the complexes shown in **A** and **B** (r.m.s.d. 1.21 Å across 303 Cα atoms). (**E**) Ribbon overlay based on the linker region and RING domain of pCBL$_{47–435}$ from the complexes shown in **A** and **B** (r.m.s.d. 0.64 Å across 74 Cα atoms). pCBL$_{47–435}$ is in the same orientation in all panels. For **C–E**, pCBL$_{47–435}$ from the UbcH5B-bound complex (**B**) is colored grey. (**F**) Images from HeLa cells overexpressing UbV.pCBL or Ub74 and treated with EGF as indicated. The cells were incubated with anti-EGFR and anti-EEA1 primary antibodies, followed by secondary antibodies conjugated to AF488 (EGFR, green) or AF594 (EEA1, red). DAPI was used to stain the nuclei. Scale bars in each panel represent 100 μm.

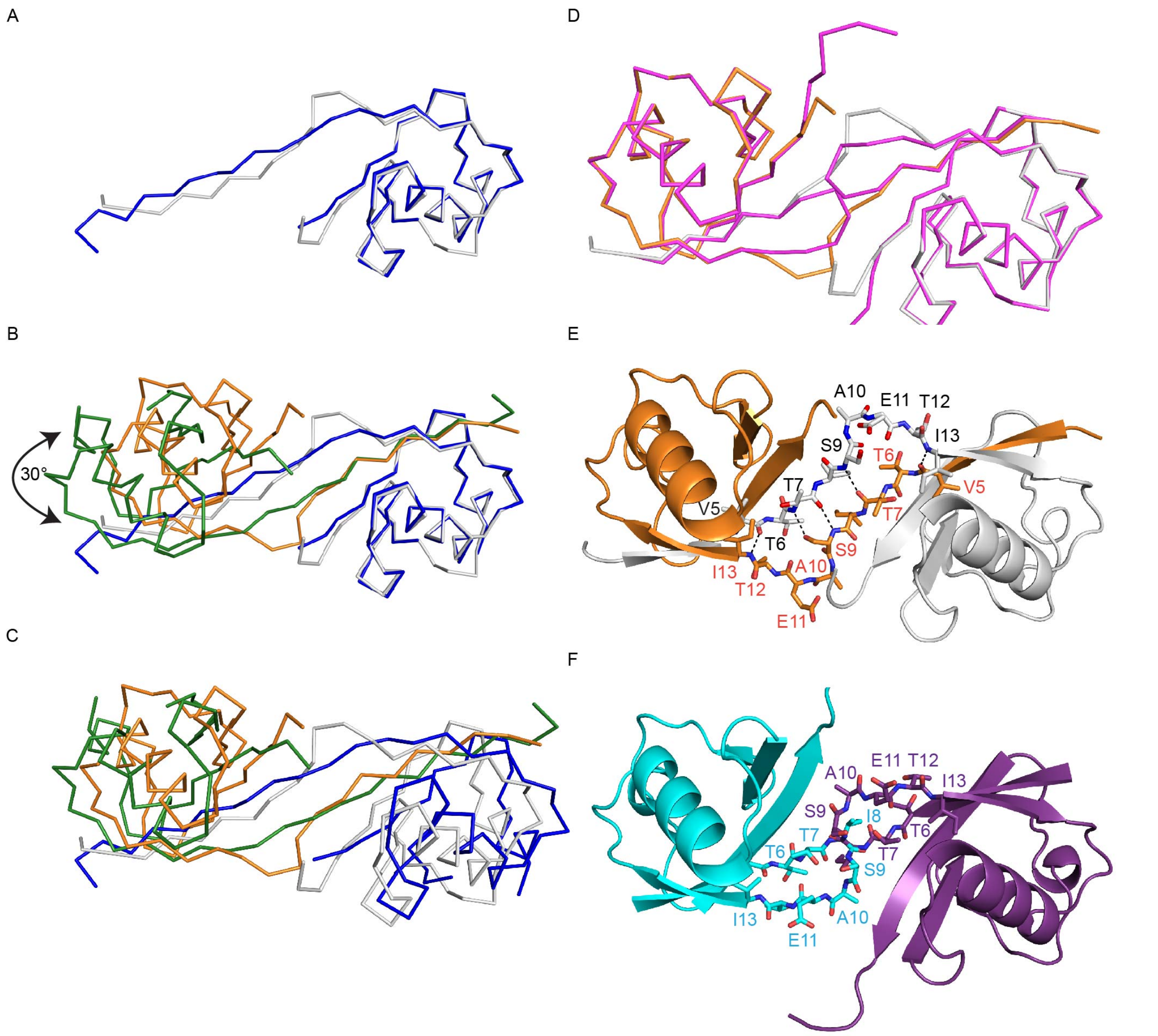

Figure S5 Comparison of UbV.XR dimers, related to Figure 5. (**A**) Ribbon overlay of a single subunit of UbV.XR$_D$ from the crystal structure of UbV.XR$_D$ alone (blue) onto UbV.XR$_D$ (white) from UbV.XR$_D$–XR complex (r.m.s.d. of 0.46 Å for 66 Cα atoms). (**B**) Ribbon overlay as in **A** but showing both subunits of UbV.XR$_D$ from the crystal structures of UbV.XR$_D$ alone (green and blue) and bound to XR (orange and white). The relative orientation of the two UbV.XR subunits differs by a 30° rotation. (**C**) Ribbon overlay of both subunits of UbV.XR$_D$ from the crystal structure of UbV.XR$_D$ alone (blue and white) onto UbV.XR$_D$ from the UbV.XR$_D$–XR complex (r.m.s.d. of 3.3 Å for 133 Cα atoms). (**D**) Ribbon overlay of Ub (magenta) onto both subunits of UbV.XR$_D$ from the UbV.XR$_D$–XR complex (r.m.s.d. of 0.4 Å for 72 Cα atoms, if β1' is treated as β1). (**E**) Close-up of UbV.XR$_D$ interface from the UbV.XR$_D$–XR complex. (**F**) Close-up of model of UbV.XR$_M$ dimer interface based on **D** in which wild-type Ub residues have been replaced with the corresponding residues in UbV.XR$_M$. One subunit is colored cyan and the other purple for clarity. Coloring is as described in **Figure 5**.

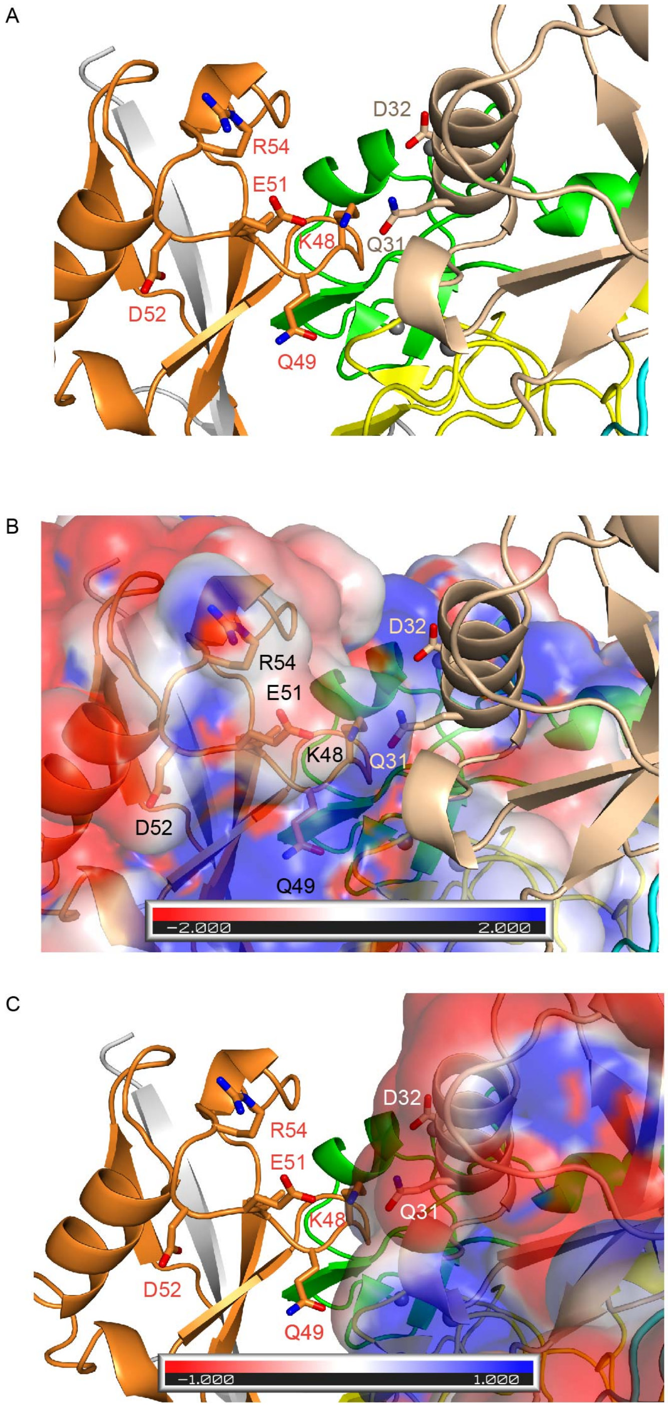

Figure S6 Mechanism of stabilization of E2~Ub by UbV.XR$_{D}$, related to Figure 5. (**A**) Close-up of donor Ub and UbV.XR$_{D}$ regions involved in electrostatic interactions from model of UbcH5B–Ub bound to the XR-UbV$_{D}$.XR complex. (**B**) As in **A** but including an electrostatic potential surface of the XR-UbV$_{D}$.XR complex. (**C**) As in **A** but including an electrostatic potential surface of UbcH5B–Ub from PDBID 4AUQ. Coloring is as described in **Figure 5**.
